# Identification of Potential Meniere's Disease Targets in the Adult Stria Vascularis

**DOI:** 10.3389/fneur.2021.630561

**Published:** 2021-02-05

**Authors:** Shoujun Gu, Rafal Olszewski, Lacey Nelson, Alvaro Gallego-Martinez, Jose Antonio Lopez-Escamez, Michael Hoa

**Affiliations:** ^1^Auditory Development and Restoration Program, National Institute on Deafness and Other Communication Disorders, National Institutes of Health (NIH), Bethesda, MD, United States; ^2^Department of Otolaryngology-Head and Neck Surgery, Georgetown University School of Medicine, Washington, DC, United States; ^3^Otology and Neurotology Group CTS495, Department of Genomic Medicine, Centre for Genomics and Oncological Research, Pfizer/Universidad de Granada/Junta de Andalucía (GENYO), Granada, Spain; ^4^Department of Otolaryngology, Instituto de Investigación Biosanitaria ibs.GRANADA, Hospital Universitario Virgen de las Nieves, Granada, Spain; ^5^Division of Otolaryngology, Department of Surgery, University of Granada, Granada, Spain

**Keywords:** stria vascularis, single-cell, nucleus, RNA-seq, Meniere's, ion homeostasis

## Abstract

The stria vascularis generates the endocochlear potential and is involved in processes that underlie ionic homeostasis in the cochlear endolymph, both which play essential roles in hearing. The histological hallmark of Meniere's disease (MD) is endolymphatic hydrops, which refers to the bulging or expansion of the scala media, which is the endolymph-containing compartment of the cochlea. This histologic hallmark suggests that processes that disrupt ion homeostasis or potentially endocochlear potential may underlie MD. While treatments exist for vestibular symptoms related to MD, effective therapies for hearing fluctuation and hearing loss seen in MD remain elusive. Understanding the potential cell types involved in MD may inform the creation of disease mouse models and provide insight into underlying mechanisms and potential therapeutic targets. For these reasons, we compare published datasets related to MD in humans with our previously published adult mouse stria vascularis single-cell and single-nucleus RNA-Seq datasets to implicate potentially involved stria vascularis (SV) cell types in MD. Finally, we provide support for these implicated cell types by demonstrating co-expression of select candidate genes for MD within SV cell types.

## Introduction

**Figure 1:**
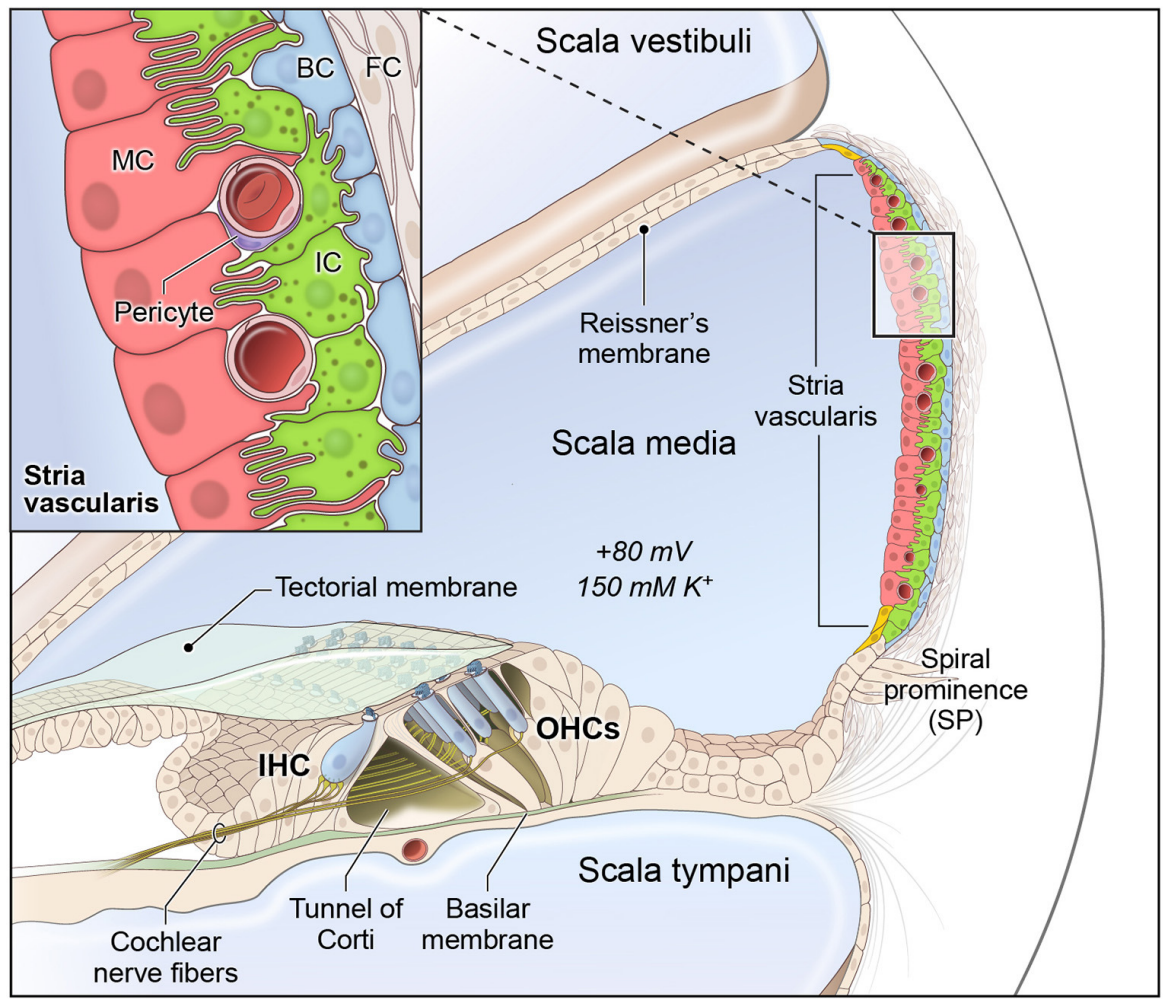
Stria vascularis cellular heterogeneity and organization. Schematic of the stria vascularis (SV) and its relationship to structures in the cochlea. The SV is composed of three layers of cells and is responsible for generating the +80 mV endocochlear potential (EP) and the high potassium concentration in the endolymph-containing scala media. The relationship between the marginal, intermediate and basal cells are demonstrated with the marginal extending basolateral projections to interdigitate with intermediate cells, which have bidirectional cellular projections that interdigitate with both marginal and basal cells. In addition to these cell types, other cell types, including spindle cells (yellow), endothelial cells, pericytes, and macrophages (not shown) are present in the SV. Used with permission from Korrapati and colleagues.

The cochlea is composed of 3 fluid-filled chambers, including the scala vestibuli, scala media, and scala tympani. Two of these chambers, the scala vestibuli and tympani, contain perilymph, which is characterized by a high sodium concentration and low potassium concentration similar to plasma in the blood. The remaining chamber, the scala media, contains endolymph, which is a special fluid characterized by a high potassium and low sodium concentration. The stria vascularis (SV) is a specialized non-sensory epithelial tissue which resides in the lateral wall of the cochlea facing the endolymph-containing scala media. The SV is composed of three main layers of cells (Figure 1), consisting predominantly of marginal, intermediate and basal cells, respectively, that function together to regulate cochlear ionic homeostasis, including potassium concentration in the endolymph, and generate the endocochlear potential (EP), which enables hair cell mechanotransduction and hearing (1–6). While the mechanisms by which both EP and cochlear ionic homeostasis are maintained remain incompletely characterized, we have recently characterized the transcriptional profiles of SV cell types and identified homeostatic gene regulatory networks using both single-cell and single-nucleus RNA-sequencing in the adult mammalian stria vascularis (7).

The histopathological hallmark of Meniere's disease (MD) is endolymphatic hydrops (EH), which refers to the expansion and bulging of the endolymph-containing scala media into the scala vestibuli as seen in processed human temporal bones (8, 9). As the stria vascularis plays a role in cochlear ionic homeostasis, the finding of EH suggests that dysfunctional ion homeostasis possibly involving the stria vascularis may result in EH and be involved in MD (10–14). Studies in human temporal bones suggest that structural changes in the stria vascularis in Meniere's disease patients exceed those in age-matched controls with presbycusis and that these structural changes correlate with observations of EH (12, 14). Despite these findings, the mechanisms underlying MD remain poorly understood and the observation of EH is thought to be an epiphenomenon or by-product of the underlying disease rather than a causal factor (8, 15). While a variety of histopathological changes in both cochlear and vestibular inner ear structures in humans have been observed, including, but not limited to, hair cells, supporting cells, the tectorial membrane, the SV and lateral wall, spiral ganglion neurons, saccule, utricle, and endolymphatic sac (9, 12–14, 16, 17), none of these findings have been definitively correlated with premortem auditory and vestibular findings (9). Furthermore, the cell types and tissues involved in the initiating pathophysiology underlying Meniere's disease remain undefined.

Despite these gaps in knowledge, a number of studies have examined genomic and transcriptomic changes in patients with MD (10, 18–32). The recent availability of single cell and single nucleus RNA-sequencing datasets from the adult mammalian stria vascularis (7) provide an opportunity to localize genes implicated in MD to relevant cell types. With this in mind, we compare the existing genomic and transcriptomic studies of MD to adult mouse SV single cell and single nucleus transcriptional profiles to identify cell types in the adult SV that express genes implicated in MD.

## Materials and Methods

### Literature Review of Meniere's Disease Implicated Genes

To provide an expanded view of the genes and variants associated with MD, we performed a systematic literature review. Facilitated by a web tool, we classified, curated, and annotated most of the genes and PubMed abstracts related to MD. Each abstract was systematically annotated for gene names and symbols. The abstracts were computationally organized by genes to be manually reviewed. After the review, the genes were classified to determine which genes showed evidence of mutations or altered expression, as defined by increased or decreased compared to control patients, in MD. In parallel, a systematic review was also performed in accordance with the Preferred Reporting Items for Systematic Reviews and Meta-analyses (PRISMA) reporting guidelines. Results of both reviews were combined to create a master list of implicated genes in Meniere's disease. The complete literature search strategy as well as study inclusion and exclusion criteria are documented in a PRISMA flowchart (Supplementary Figure 1).

#### PubTerm Search Strategy

To obtain unbiased updated information on genes with mutations or altered expression in MD, we acquired PubMed abstracts related to reported polymorphisms or altered expression in MD in August 2020 using a web tool called PubTerm (http://bioinformatica.mty.itesm.mx:8080/Biomatec/pubterm.html) to initially organize, annotate, and curate abstracts per gene (33–35). Briefly, the PubTerm tool organizes abstracts by genes, chemicals, diseases, species, and other terms, or by the co-occurrences of genes and diseases that facilitate classification, annotation, curation, and tracking of genes of interest. We used the following query terms: (“Meniere's Disease”[TIAB] OR “Meniere disease” OR “endolymphatic hydrops”[TIAB]) AND (mutations[TIAB] OR mutation[TIAB] OR polymorphisms[TIAB] OR polymorphism[TIAB] OR variant[TIAB] OR variants[TIAB] OR RNA-sequencing[TIAB]) NOT review[Publication Type]. Addition of the term “GWAS” or “genome-wide association study” did not change the number of references identified. Subsequently, full text reviews of all identified references were performed to identify genes not mentioned in the abstract and to include gene candidates provided in datasets that accompanied references where applicable.

#### PRISMA Search Strategy

The following databases and gray literature sources were searched from inception through August 1, 2020: PubMed-NCBI, MEDLINE, Embase, CINAHL, Cochrane Library, ClinicalTrials.gov, OpenGrey, GreyNet, GreyLiterature Report, and European Union Clinical Trials Registry. No language restriction was employed. Previously described search terms were utilized. Article titles and abstracts were screened for eligibility before full-text articles were obtained and assessed for possible inclusion. Additional relevant articles were identified through the reference lists of included studies. References identified by PubTerm were also incorporated into the PRISMA strategy and screened as described (36). The final reference list was reviewed independently by two reviewers. Data from included studies was extracted and compiled in a standardized electronic data collection sheet. The primary outcome of interest was the gene(s) or genetic polymorphism(s) of interest being investigated.

### Animals

Inbred CBA/J males and females were purchased from JAX (Stock No. 000656). Breeding pairs were set up to obtain P30 mice for immunohistochemistry and single molecule RNA FISH.

### Single Molecule Fluorescent *in situ* Hybridization (smFISH) and Immunohistochemistry

#### smFISH Using RNAscope Probes

Briefly, *in situ* hybridizations were performed as previously described (7). The following RNAscope probes were utilized: *Kcne1* (Cat# 541301), *Atp1b2* (Cat# 417131), *Esrrb* (Cat# 565951-C3), *Met* (Cat# 405301), *Ednrb* (Cat# 473801-C3), and *Tmem176a* (Cat# 432641-C2). RNAscope probes were obtained from Advanced Cell Diagnostics (Newark, CA) and used with sections of cochleae from CBA/J wild type mice at P30. Adult cochleae were dissected from the head and fixed overnight at 44°C in 4% PFA in 1x PBS. Fixed adult mouse inner ears were decalcified in 150 mM EDTA for 5–7 days, transferred to 30% sucrose, and then embedded and frozen in SCEM tissue embedding medium (Section-Lab Co, Ltd.). Adhesive film (Section-Lab Co, Ltd.; Hiroshima, Japan) was fastened to the cut surface of the sample in order to support the section and cut slowly with a blade to obtain thin midmodiolar sections. The adhesive film with section attached was submerged in 100% EtOH for 60 s, then transferred to distilled water. The adhesive film consists of a thin plastic film and an adhesive and it prevents specimen shrinkage and detachment. This methodology allows for high quality anatomic preservation of the specimen. Frozen tissues were sectioned (10 μm thickness) with a CM3050S cryostat microtome (Leica, Vienna, Austria). Sections were mounted with SCMM mounting media (Section-Lab Co, Ltd., Hiroshima, Japan) and imaged using a 1.4 N.A. objective. Labeling with 4,6-diamidino-2-phenylindole (DAPI, 1:10,000, Life Technologies) was included to detect cell nuclei.

### Bioinformatics

#### Data and Software Availability

Previously published single cell and single nucleus RNA-Seq datasets of postnatal day 30 (P30) mouse stria vascularis (7) were utilized (GEO Accession ID: GSE136196) which can be found at the following link (https://www.ncbi.nlm.nih.gov/geo/query/acc.cgi?acc=GSE136196) and are available through the gene Expression Analysis Resource (gEAR), a website for visualization and comparative analysis of multi-omic data, with an emphasis on hearing research (https://umgear.org//index.html?layout_id=b50cae7a) (37).

#### Data Visualization

##### P30 SV scRNA-Seq & snRNA-Seq

Previously published P30 SV scRNA-Seq and snRNA-Seq data were preprocessed by Scanpy (v1.5.1) with criteria as previously described (7). Briefly, low-quality and outlier cells were computationally removed if: (1) gene number per cell or nuclei was less than the 5th percentile or more than 95th percentile; (2) total counts per cell or nuclei was less than the 5th percentile or more than 95th percentile; (3) >20% mitochondria genes (snRNA-Seq only). Predicted doublets by Scrublet (v0.2.1) with default settings were also filtered.

Preprocessed data were normalized by total with parameter *exclude_highly_expressed* set as “True” and scaled by the function *pp.log1p()*. Cell clustering and annotation was performed using modularity-based clustering with Leiden algorithm implemented in Scanpy (v1.5.1). Heatmap were plotted by Seaborn (v0.10.1). Dotplots were plotted by the Scanpy function *pl.dotplot(*).

#### Gene Ontology and Gene Set Enrichment Analysis

Gene ontology analyses and gene set enrichment analyses were performed using Enrichr (http://amp.pharm.mssm.edu/Enrichr/) as previously described (7, 38–41). The combined score approach where enrichment score is calculated from the combination of the *p*-value computed using the Fisher exact test and the z-score was utilized. Top gene ontology (GO) terms were chosen by utilizing the combined score approach as described. Genes were further functionally classified using the Protein Analysis Through Evolutional Relationships (PANTHER, pantherdb.org) database (42).

## Results

### Systematic Literature Reviews Curate Genes Implicated in Meniere's Disease

In order to curate a comprehensive list of genes implicated in MD, we utilized parallel methodologies for systematic reviews including the use of a webtool, PubTerm, to perform an unbiased search for implicated genes using the previously noted search terms in PubMed and a manual search of public databases and gray literature using PRISMA guidelines. In total, 389 unique abstracts were identified. Abstracts unrelated to Meniere's disease (*n* = 196) were excluded resulting in 193 abstracts related to Meniere's disease. Exclusion criteria including non-English language (*n* = 22), animal studies (*n* = 21), non-gene outcome defined as an absence of genes studied in relation to Meniere's disease (*n* = 45), and unrelated to Meniere's disease (*n* = 28) were applied to a full-text review of these references resulting in 77 references being included for systematic review (Supplementary Figure 1). Full-text reviews of identified reference articles as well as review of attached datasets were utilized to ensure that a comprehensive list of genes was defined from these references. Based on the described search strategy, a total of 832 genes were investigated in relation to MD, and 122 of the genes were reported in more than one study. A table of the identified genes and their references is included (Supplementary Table 1). We have provided descriptive tables summarizing the studies implicating genes in MD (Supplementary Datas 1, 2).

**Figure 2:**
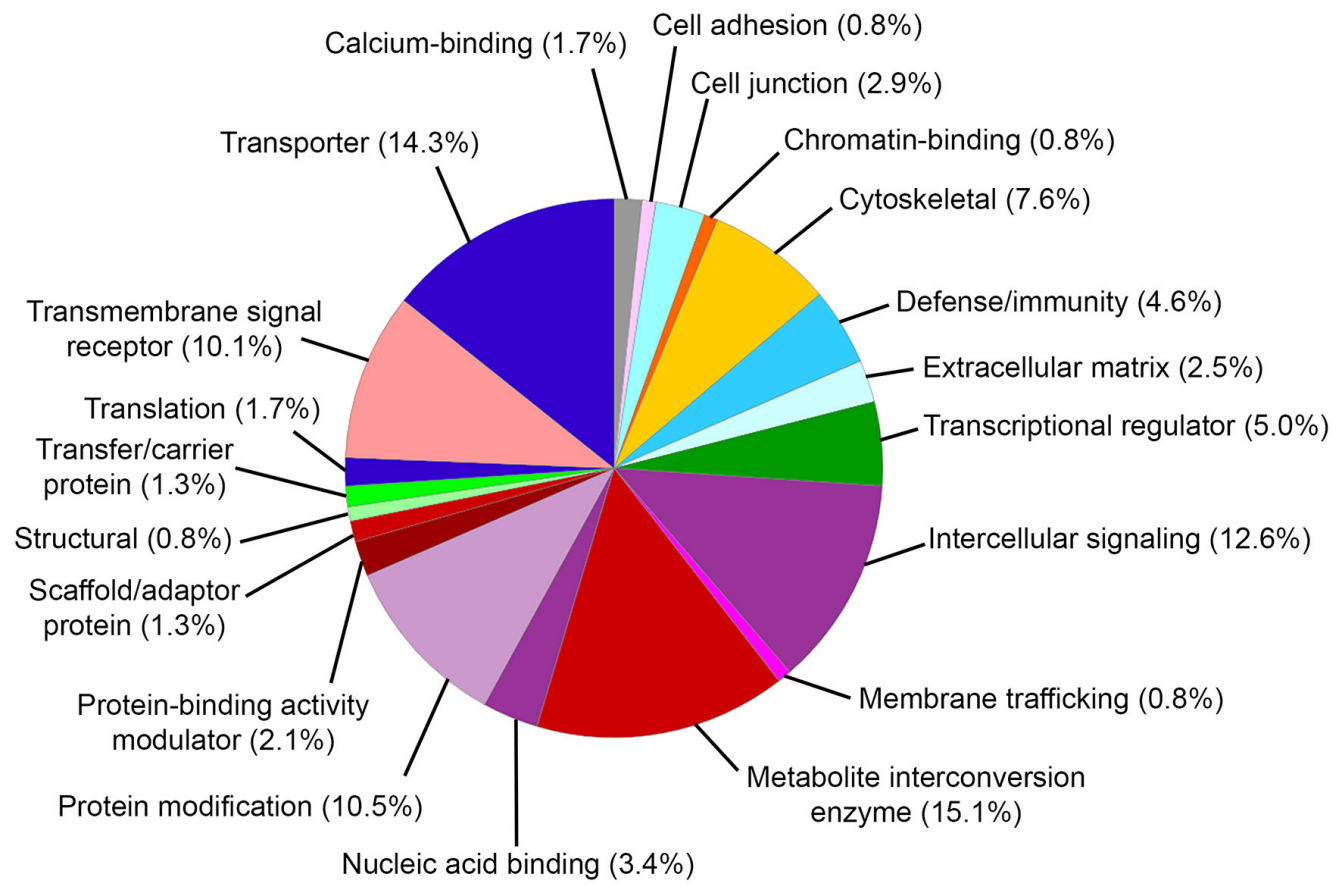
PANTHER protein classification of genes implicated in Meniere's disease. Pie chart depicts protein classes found in gene list. Percentages reflect number of gene hits against total number of process hits. The top protein classes include metabolite interconversion enzymes, transporters, intercellular signaling proteins, protein modification proteins, and transmembrane signaling receptors.

Gene ontology (GO) biological process analysis of these genes implicated in MD identified significant enrichment for cellular metal ion homeostasis (GO:0006875), positive regulation of calcium ion transport into cytosol (GO:0010524), positive regulation of calcium ion transmembrane transport (GO:1904427), equilibrioception (GO:0050957), sensory perception of mechanical stimulus (GO:0050954), and sensory perception of sound (GO:0007605). GO molecular function analysis identified significant enrichment for cytokine activity (GO:0005125), water transmembrane transporter activity (GO:0005372), water channel activity (GO:0015250), chemokine activity (GO:0008009), chemokine receptor binding (GO:0042379), and sodium ion transmembrane transporter activity (GO:0015081). GO cellular component analysis identified significant enrichment for integral component of plasma membrane (GO:0005887), MHC protein complex (GO:0042611), and junctional sarcoplasmic reticulum membrane (GO:0014701). The PANTHER classification identified 20 functional groups and revealed that genes implicated in MD encoded metabolite interconversion enzymes (15.1%, including *Mif* and *Sod2*), transporters (14.3% including *Atp1b2* and *Trpv4*), intercellular signaling proteins (12.6%, including *Tgfb2*), protein modification proteins (10.5%, including *Wnk2, Wnk4*, and *Sgk1*), transmembrane signaling receptors (10.1%, including *Adrb2*), and others (Figure 2).

### Single-Cell and Single-Nucleus RNA-Sequencing of the Adult Mouse Stria Vascularis Reveal Expression of Genes Associated With MD in Major SV Cell Types

**Figure 3:**
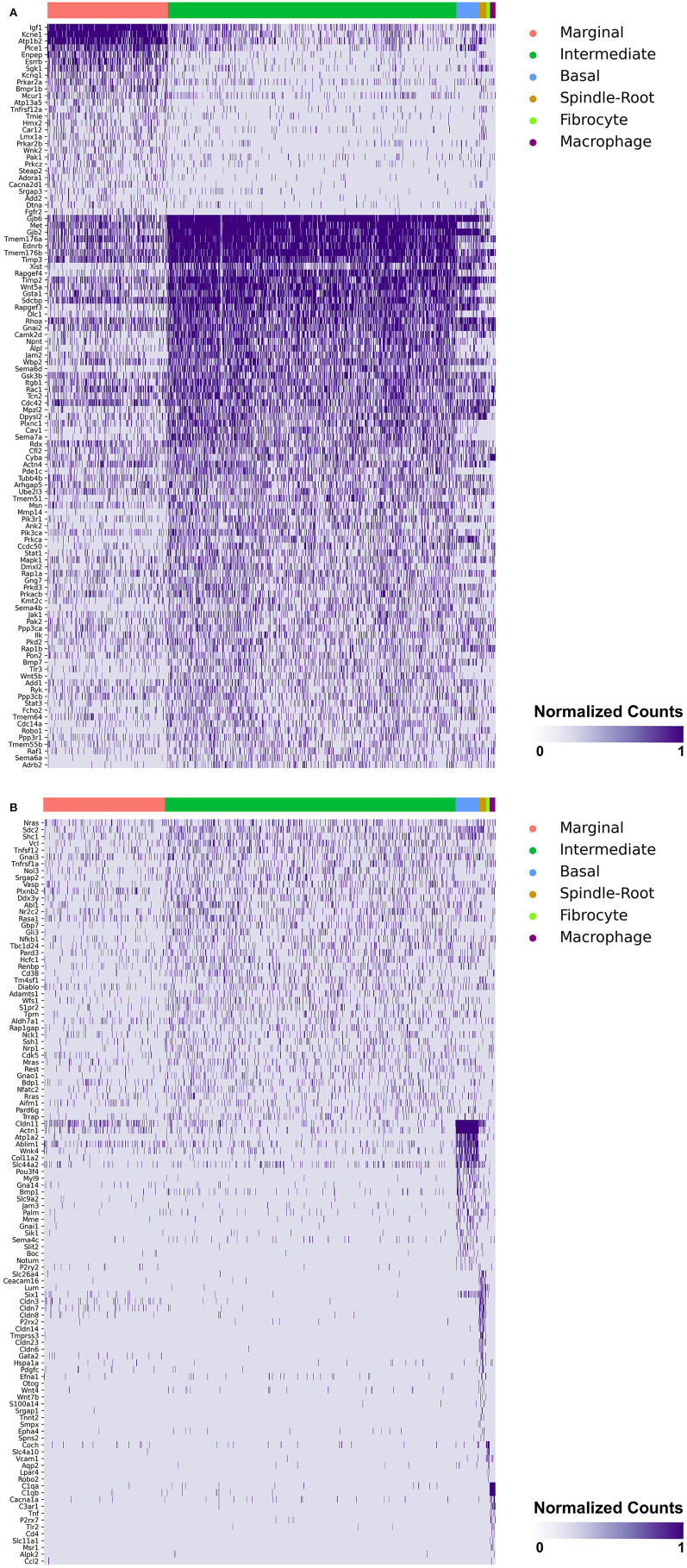
**(A)** SV cell type-specific expression of genes implicated in Meniere's disease as demonstrated by single-cell RNA-seq: marginal & intermediate cells. Heatmap displays genes with cell type-specificity predominantly to marginal and intermediate cells, respectively. Heatmap displays cell types along the horizontal axis and genes along vertical axis. Gene expression is displayed in normalized counts. Cell types displayed include marginal cells, intermediate cells, basal cells, spindle-root cells, fibrocytes, and macrophages. **(B)** SV cell type-specific expression of genes implicated in Meniere's disease as demonstrated by single-cell RNA-seq: intermediate cells, basal cells, spindle-root cells, fibrocytes, and macrophages. Heatmap displays genes with cell type-specificity predominantly to intermediate, basal cells, spindle-root cells, fibrocytes, and macrophages, respectively. Heatmap displays cell types along the horizontal axis and genes along vertical axis. Gene expression is displayed in normalized counts. Cell types displayed include marginal cells, intermediate cells, basal cells, spindle-root cells, fibrocytes, and macrophages.

**Figure 4:**
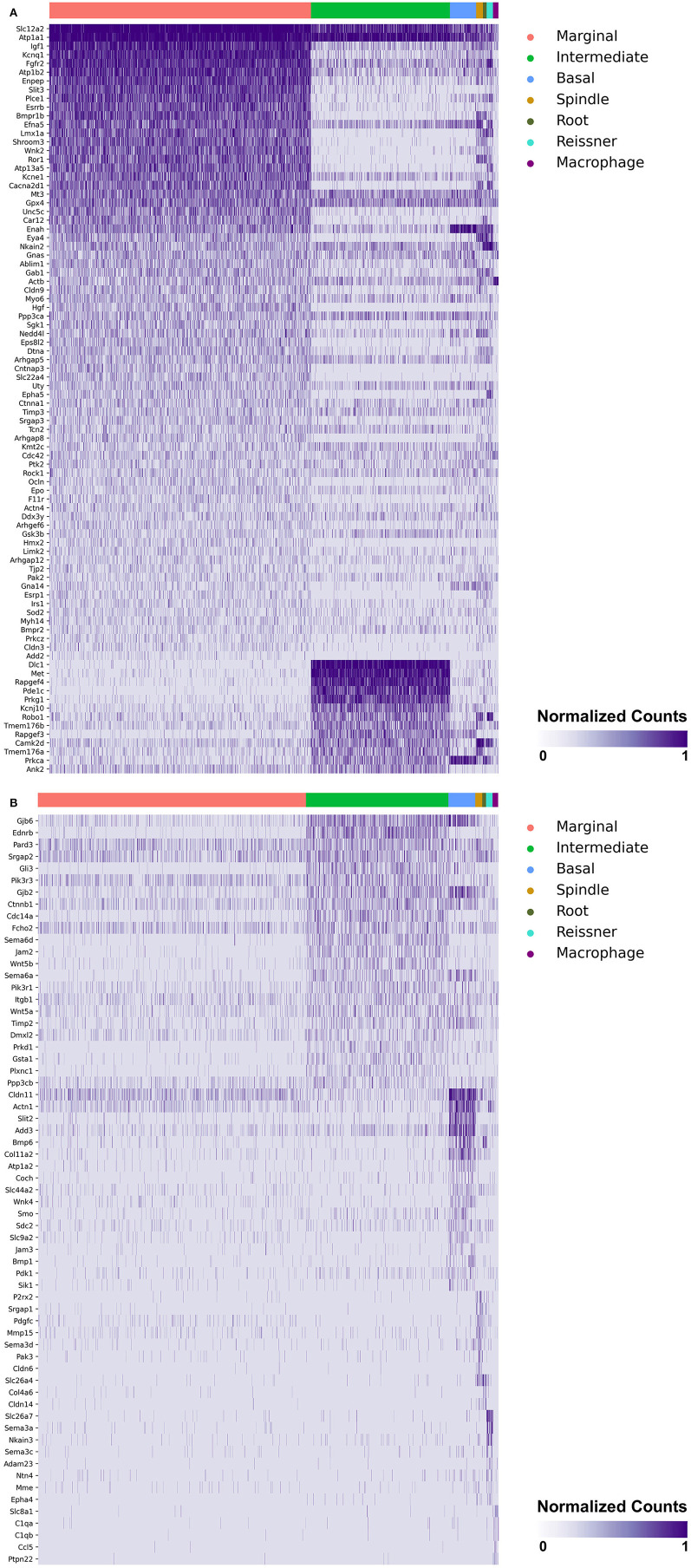
**(A)** SV cell type-specific expression of genes implicated in Meniere's disease as demonstrated by single-nucleus RNA-seq: marginal & intermediate cells. Heatmap displays genes with cell type-specificity predominantly to marginal and intermediate cells, respectively. Heatmap displays cell types along the horizontal axis and genes along vertical axis. Gene expression is displayed in normalized counts. Cell types displayed include marginal cells, intermediate cells, basal cells, spindle cells, root cells, Reissner's membrane cells, and macrophages. **(B)** SV cell type-specific expression of genes implicated in Meniere's disease as demonstrated by single-nucleus RNA-seq: intermediate cells, basal cells, spindle cells, root cells, Reissner's membrane cells, and macrophages. Heatmap displays genes with cell type-specificity predominantly to intermediate cells, basal cells, spindle cells, root cells, Reissner's membrane cells, and macrophages, respectively. Heatmap displays cell types along the horizontal axis and genes along vertical axis. Gene expression is displayed in normalized counts. Cell types displayed include marginal cells, intermediate cells, basal cells, spindle cells, root cells, Reissner's membrane cells, and macrophages.

Heatmaps demonstrating genes reported in MD with cell type specificity in both the single-cell and single-nucleus RNA-sequencing datasets are shown in Figures 3A,B (single-cell RNA-Seq) and Figures 4A,B (single-nucleus RNA-Seq), respectively. In the single-cell RNA-seq dataset, fibrocytes were detected and the number of spindle and root cells captured did not enable their transcriptional profiles to be distinguished. In the single-nucleus RNA-seq dataset, spindle and root cell transcriptional profiles were distinguishable, Reissner's membrane cells were detected, and fibrocytes were not detected. Stria vascularis from adult mice were collected, attempting to remove as much of the spiral ligament from the strial tissue. Fibrocytes that were detected represent contaminating cells from the spiral ligament. In both datasets, marginal cells, intermediate cells, basal cells, macrophages, B cells and neutrophils were detected. Details related to these datasets have been described previously (7). Heatmaps demonstrating expression of genes without cell type-specific expression are shown in the supplement for single-cell RNA-seq (Supplementary Figures 2–4) and single-nucleus RNA-Seq (Supplementary Figures 5–9), respectively.

**Figure 5:**
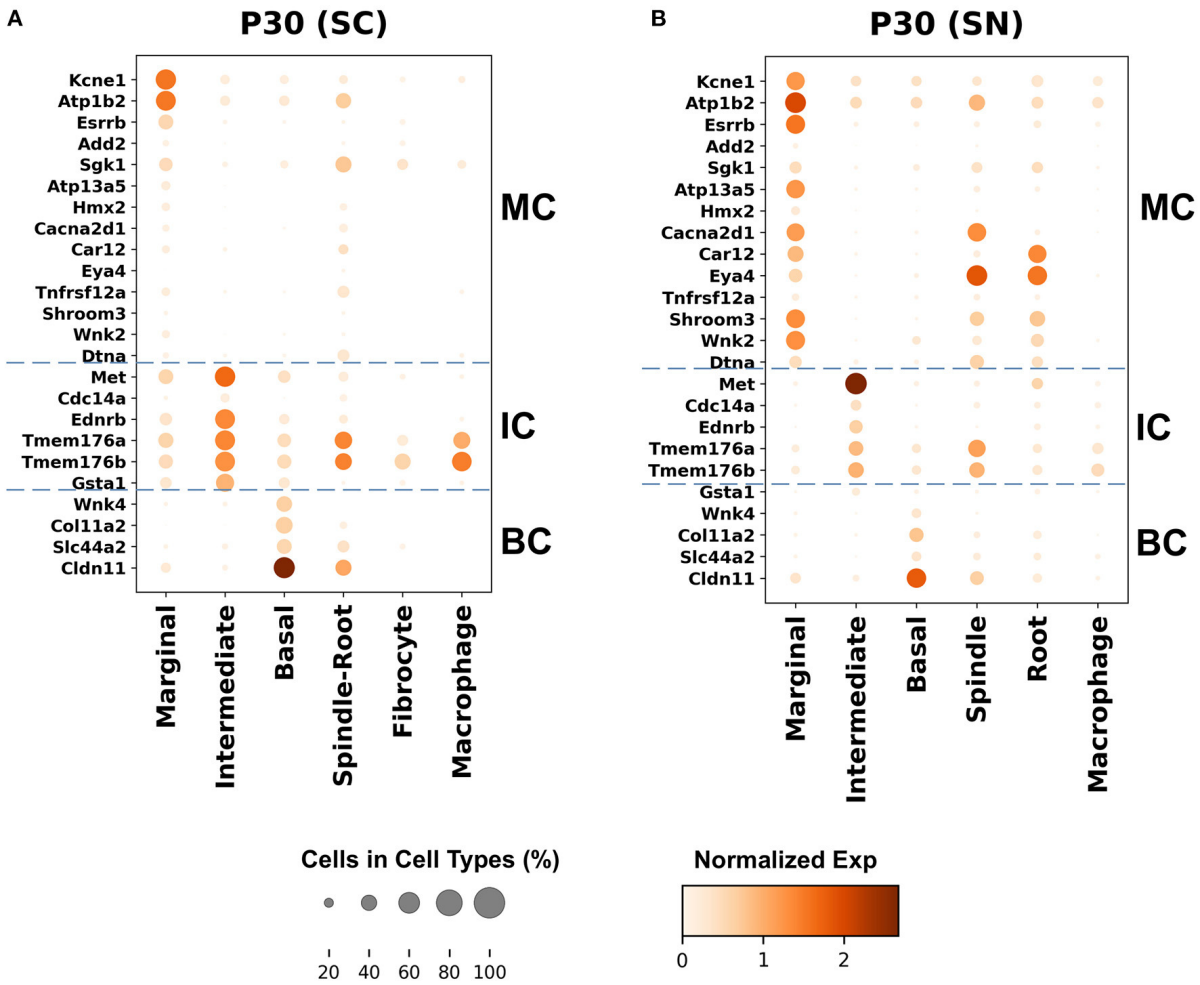
Top differentially expressed Meniere's disease genes in the adult SV. **(A)** Top differentially expressed genes in the single-cell RNA-seq dataset. **(B)** Top differentially expressed genes in the single-nucleus RNA-seq dataset. Cell types are displayed along the horizontal axis and genes are displayed along the vertical axis. The size of the dot corresponds to the proportion of cells expressing the given gene (see legend). The darker the color of the dot, the greater the level of expression of the given gene (see scale bar).

Genes with preferential expression in SV marginal cells include *Kcne1, Atp1b2, Esrrb, Add2, Sgk1, Atp13a5, Hmx2, Cacna2d1, Car12, Eya4, Tnfrsf12a, Shroom3, Wnk2*, and *Dtna* (Figures 3A, 4A). Dot plots demonstrate differential expression amongst the major strial cell types including marginal, intermediate and basal cells, as well as some rarer cell types, including SV spindle and root cells, and SV macrophages (Figures 5A,B). In each dot plot, the more orange the dot, the greater the expression level of a given gene and the larger the dot, the greater the proportion of cells that express the given gene. Genes with previously published expression in marginal cells, include *Kcne1* (2, 43), *Atp1b2* (2, 44–47), *Esrrb* (7, 48), and *Atp13a5* (7). Examination of SGK1 protein expression in the rat and guinea pig cochlea demonstrates expression in the stria vascularis in addition to the spiral ganglion neurons, spiral limbus, organ of corti, and spiral ligament (49, 50). Examination of *in situ* hybridization of *Sgk1* in the E15.5 mouse in the Allen Brain Atlas suggests localization to the organ of Corti and the roof of the cochlear duct where future marginal cells reside (Supplementary Figure 10A). *Hmx2* has been previously localized to the developing mouse stria vascularis (51). *Cacna2d1* has been previously localized to spiral ganglion neurons (52) and examination of *in situ* hybridization in the E15.5 mouse in the Allen Brain Atlas suggests possible localization to the roof of the cochlear duct where future marginal cells reside (Supplementary Figure 10B). Carbonic anhydrases have been previously shown to be expressed in the stria vascularis (53–55) and *Car12* expression has been previously shown to be expressed in root cells with reduced expression in marginal cells (56). *Eya4* has not been previously localized to the mouse stria vascularis but examination of *in situ* hybridization in the E15.5 mouse in the Allen Brain Atlas suggests widespread expression in the cochlear duct including the region of the future stria vascularis (Supplementary Figure 10C). *Add2, Tnfrsf12a, Shroom3, Wnk2*, and *Dtna* have not been previously localized to structures in the cochlea.

Genes with preferential expression in SV intermediate cells include *Met, Cdc14a, Ednrb, Tmem176a, Tmem176b*, and *Gsta1* (Figures 3A, 4A). Dot plots demonstrate differential expression amongst marginal, intermediate, and basal cells in the SV (Figures 5A,B). *Met* has been previously shown to be expressed in intermediate cells in the developing and adult mouse SV (7, 57). *Cdc14a* has been previously shown to be expressed in hair cell stereocilia and hair cells, supporting cells, the osseous spiral lamina, and spiral ganglion neurons (58). *Ednrb* has been previously localized to the SV but the specific cell type expressing *Ednrb* could not be defined (59). *Tmem176a, Tmem176b*, and *Gsta1* have not been previously localized to structures in the cochlea.

Genes with preferential expression in basal cells within the SV include *Wnk4, Col11a2*, and *Slc44a2* (Figures 3B, 4B). Dot plots demonstrate differential expression amongst marginal, intermediate, and basal cells in the SV (Figures 5A,B). These genes are co-expressed by SV basal cells which are identified by *Cldn11* expression that has been previously demonstrated in both adult mouse and humans (7, 60, 61). *Col11a2* has been previously shown to be expressed in the spiral ligament (SL) and has been associated with a variety of syndromic as well as non-syndromic sensorineural hearing loss including both autosomal-dominant (DFNA13) and autosomal-recessive (DFNB53) forms (62–64). Slc44a2, formerly known as choline transporter-like protein 2 (CTL-2), is a multi-transmembrane protein originally discovered as a target of antibody-induced hearing loss (65) and more recently implicated in Meniere's disease (28). Others have suggested that because of its prominent expression in cells facing the scala media, SLC44A2 may play a role in cochlear ionic homeostasis (65). *Wnk4* is a known regulator of claudins and paracellular chloride ion permeability (66–68). *Wnk4* has not been previously localized to SV basal cells. In summary, these data demonstrate RNA expression of Meniere's candidate genes in adult mouse SV cell types.

### Single Molecule Fluorescent *in situ* Hybridization (smFISH) Localizes Genes Implicated in Meniere's Disease to the Stria Vascularis

**Figure 6:**
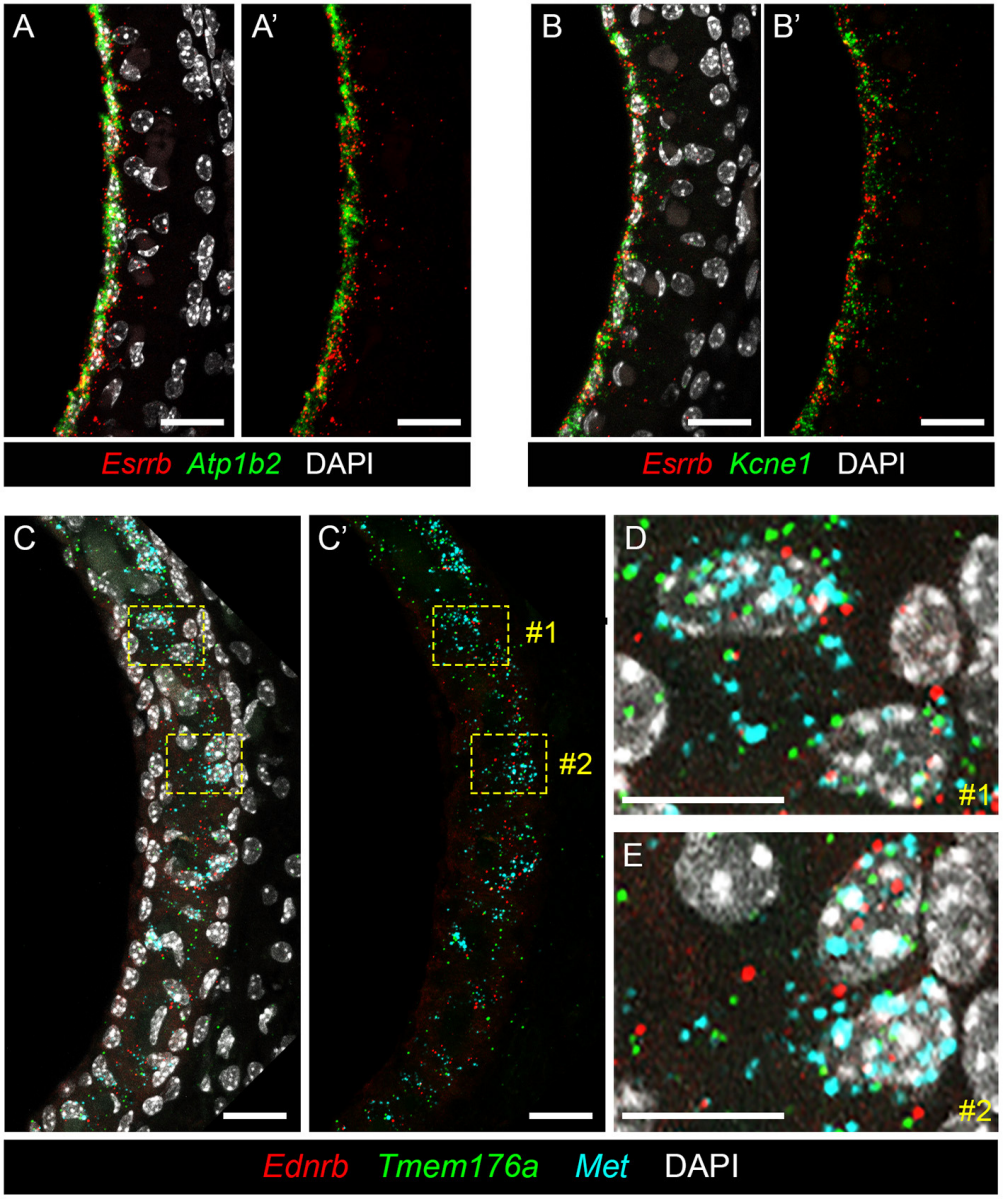
Validating expression of genes implicated in Meniere's disease in the adult mouse SV. **(A,A′ )** RNA probes demonstrate co-expression of *Esrrb* (red) and *Atp1b2* (green) RNA in marginal cells of the adult mouse SV (P30 mouse) with DAPI (white) labeling of cell nuclei **(A)** and without DAPI labeling of cell nuclei **(A′ )**. Scale bars are 20 microns. **(B,B′ )** RNA probes demonstrate co-expression of *Esrrb* (red) and *Kcne1* (green) RNA in marginal cells of the adult mouse SV (P90 mouse) with DAPI (white) labeling of cell nuclei **(B)** and without DAPI labeling of cell nuclei **(B′ )**. Scale bars are 20 microns. **(C,C′ )** RNA probes demonstrate co-expression of *Met* (turquoise), *Ednrb* (red) and *Tmem176a* (green) RNA in the intermediate cell layer of the adult mouse SV (P90 mouse) with DAPI labeling of cell nuclei **(C)** and without DAPI labeling of cell nuclei **(C′ )**. Scale bars are 20 microns. Yellow dashed line boxes outline representative cells where the RNA signal of all 3 probes are colocalized to intermediate cell nuclei. **(D,E)** Closeup images of representative cells #1 **(D)** and #2 **(E)** from **(C,C′ )** where RNA expression of *Met* (turquoise), *Ednrb* (red), and *Tmem176a* (green) are co-expressed in intermediate cell nuclei. Scalebars are 10 microns.

To localize genes implicated in Meniere's disease to the stria vascularis, we performed smFISH. Having previously validated expression of *Esrrb* RNA in adult SV marginal cells (7), we demonstrate co-expression of this Meniere's disease-implicated gene with *Atp1b2* (Figures 6A,A′) and *Kcne1* (Figures 6B,B′) in adult mouse SV marginal cells. The RNA of both *Atp1b2* (Figure 6A) and *Kcne1* (Figure 6B) localizes predominantly to the marginal cell nuclei. Images without DAPI labeling are shown to emphasize the co-localization of *Esrrb* with *Atp1b2* (Figure 6A′) and *Kcne1* (Figure 6B′), respectively. Missense mutations in *ESRRB* have been identified in patients with Meniere's disease (27) as well as an autosomal recessive non-syndromic sensorineural hearing loss DFNB35 (48). *ATP1B2* is upregulated in peripheral blood mononuclear cells (PBMCs) obtained from patients with Meniere's disease compared to patients without Meniere's disease (23). Single nucleotide polymorphisms (SNPs) in KCNE1 have been identified in patients with Meniere's disease (29, 69–72). However, Campbell and colleagues, in comparing two larger cohorts of Meniere's disease and control patients in the Caucasian population, failed to find a significant association between several KCNE1 SNPs and Meniere's disease (73). In intermediate cells, we co-localize expression of *Met, Ednrb*, and *Tmem176a* RNA (Figures 6C–E). *Met* (in turquoise), *Ednrb* (in red), and *Tmem176a* (in green) RNA are localized to the intermediate cell layer of the stria vascularis (Figure 6C). Image without DAPI labeling (Figure 6C′) is shown to emphasize co-expression of *Ednrb, Tmem176a*, and *Met* in the intermediate cell layer. Yellow dashed line boxes delineate two representative regions (region #1 and region #2) that are enlarged to serve as representative examples of *Ednrb, Tmem176a*, and *Met* RNA co-localizing to intermediate cell nuclei within the SV (Figures 6D,E, respectively). While SNPs of uncertain significance for *MET* were identified in patients with familial Meniere's disease (22), PBMCs from Meniere's patients demonstrate increased expression of both *EDNRB* and *TMEM176A* compared to control patients (23). These examples validate the ability of these datasets to localize genes implicated in Meniere's disease to specific cell types in the adult stria vascularis.

## Discussion

Meniere's disease may represent multiple disease entities with a common set of presenting symptoms. The identification of a heterogeneous group of mutations in human genes with variable expressivity within familial MD cohorts (74) and an increased burden of rare missense variants in several SNHL genes (27) and axonal guidance signaling genes (24) in sporadic MD in the Spanish population serve as examples of evidence that support this contention. Given this suspected heterogeneity, single-cell and single-nucleus transcriptional datasets offer the opportunity to localize genes identified in poorly understood diseases like MD to involved cell types and tissue structures. Gene ontology analysis of systematically curated genes implicated in MD suggests that these genes may play a role in ion homeostasis and immune function and functional analysis suggests that these genes are involved in metabolism, transport, intercellular and transmembrane cell signaling, and protein processing. While possibly not surprising given that ion homeostatic dysfunction and immune dysfunction (10, 25, 30, 75) have been postulated mechanisms underlying MD likely motivating at least some of the reviewed studies, this review utilizes an integrative approach to derive a top-down perspective on genes implicated in MD. More importantly, we utilize these systematically curated genes implicated in MD and single-cell transcriptional profiles from the adult mouse SV to localize these genes to the adult stria vascularis and for the first time, implicate SV cell types in the underlying pathophysiology of MD. Based on these results, we suggest that these data have several implications.

### Localization of Genes Implicated in Meniere's Disease to Major SV Cell Types Suggests That Dysfunction in These Cell Types May Contribute to Mechanisms Underlying Meniere's Disease

First, the larger number of genes expressed by marginal and intermediate cells in the SV suggests that these cells may play a more prominent role in MD. The global localization of these implicated genes to major SV cell types is supported by both our previous work in localizing *Kcne1, Atp13a5*, and *Esrrb* RNA to marginal cells, *Met* RNA to intermediate cells, and *Slc26a4* and *P2rx2* RNA to spindle cells (7) as well as newly localized gene candidates to major adult SV cell types, including *Ednrb* and *Tmem176a* (Figure 5). Connexin 26 (GJB2) and Connexin 30 (GJB6) protein have been previously localized to SV intermediate and basal cells in humans (76, 77) and in mouse (78). While localization of expression does not necessarily imply functional significance, the localization of many genes to specific cell types in the SV may suggest that understanding the roles of these cell types may be critical for insight into the underlying pathophysiology of MD.

### Implications for the Development of Mouse Models of Meniere's Disease

In localizing Meniere's disease genes to major SV cell types, we suggest that this points to the need to better understand the role that dysfunction in each of these cell types plays in hearing instability and loss. Work by Gallego-Martinez and colleagues suggests the possibility that the accumulation of missense variants in some which include genes expressed by SV cell types may contribute to the development of MD (27). Meniere's disease has an onset of disease in adulthood (79–81) and this suggests a need for inducible mouse models of gene dysfunction which would distinguish the effects of gene dysfunction on hearing in the mature cochlea from those related to dysfunction during development. More generally, mouse models that replicate hearing fluctuation seen in human patients with MD could serve as useful pre-clinical models to examine the mechanism and efficacy of repurposed and novel therapeutics for MD.

### Implications for Mechanisms Underlying Meniere's Disease

Furthermore, investigating dysfunctional calcium homeostasis may be important to understanding mechanisms underlying hearing loss in MD. Gene ontology analysis of genes implicated in MD identified mechanisms involving calcium ion transport as being enriched. Of these genes, single-cell and single-nucleus RNA-Seq datasets demonstrate expression of *Atp13a5, Cacna2d1*, and *Trpv4* in SV marginal cells, *Ank2, Cav1*, and *Wfs1* in SV intermediate cells, and *P2rx2* in SV spindle cells. Of these genes, evidence of decreased expression in MD has been noted for *Atp13a5, Cacna2d1*, and *Ank2* (23) while *Cav1* expression has been shown to be increased in patients with MD (23, 31). While it has been suggested that *TRPV4* expression may be decreased in human endolymphatic sac tissue from MD patients (82), a subsequent case-control replication study examining *TRPV4* expression failed to demonstrate an association between *TRPV4* expression and MD (83). Expression of *TRPV4* in the cochlea in patients with MD has not been compared to unaffected human patients. Missense variants of uncertain significance for *WFS1* were identified in patients with MD but a significant excess of these variants was not seen when compared to control populations (27). In contrast, an excess of *P2RX2* missense variants was noted in patients with MD (27). In an experimental model for endolymphatic hydrops, Salt and DeMott have previously demonstrated that elevations in calcium concentration in the endolymph, at a time when endolymph volume and the EP are no longer changing, correlates with elevated auditory thresholds (84). These authors suggest that the gradual dysregulation of calcium concentration in the endolymph while associated with endolyphatic hydrops, may be the underlying mechanism of hearing loss in these settings. Providing further evidence to support this theory, Wangemann and colleagues have shown that the loss of *Slc26a4* expression in a mouse model for Pendred syndrome results in acidification of the endolymph, a failure of calcium reabsorption from the endolymph, and hearing loss (85). Thus, these data suggest that dysfunctional calcium homeostasis within the endolymph may potentially contribute to mechanisms resulting in the development of MD.

### Limitations

Despite these observations, several caveats apply to the data presented. A large proportion of gene expression changes for genes implicated in MD were determined from expression changes in the peripheral blood mononuclear cells, which may not reflect changes in the inner ear. Furthermore, genomic features, including single nucleotide variants, copy number variants, and other structural variants have not been systematically and uniformly examined in relation to these investigated genes, potentially contributing to bias in the interpretation of the genomic underpinnings of Meniere's disease. Nonetheless, we localize these genes implicated in MD to the SV providing a context for beginning to understand these observed changes and identifying potentially relevant inner ear gene targets. Furthermore, identifiable expression does not necessarily equate to functional importance. Finally, while we acknowledge the limited ability to connect functional attribution of broad expression changes in the blood to mechanisms underlying MD, identifying meaningful gene and cellular targets in the SV establishes a basis for testing hypotheses related to the underlying pathophysiology of MD.

In conclusion, utilizing single-cell and single-nucleus transcriptional profiles, we localize genes implicated in MD to adult stria vascularis cell types. We identify trends in potentially involved SV cell types based on this top-down approach and in doing so, provide justification for the development of inducible cell type-specific models of SV dysfunction as a means of investigating the underlying pathophysiology of MD. Finally, we provide evidence of the reliable ability of our published transcriptional profiles to localize several candidate gene targets to specific SV cell types, establishing a justification for testing the role of these candidate genes in MD.

## Data Availability Statement

Publicly available datasets were analyzed in this study. This data can be found at: Previously published single cell and single nucleus RNA-Seq datasets of postnatal day 30 (P30) mouse stria vascularis (7) were utilized (GEO Accession ID: GSE136196) which can be found at the following link (https://www.ncbi.nlm.nih.gov/geo/query/acc.cgi?acc=GSE136196) and are available through the gene Expression Analysis Resource (gEAR), a website for visualization and comparative analysis of multi-omic data, with an emphasis on hearing research (https://umgear.org//index.html?layout_id=b50cae7a) (37).

## Author Contributions

SG and MH contributed to bioinformatic analysis of previously published scRNA-Seq and snRNA-Seq datasets. RO and MH were responsible for smFISH and immunohistochemistry. LN performed systematic review of Meniere's disease-implicated genes. SG, RO, LN and MH contributed to primary draft of manuscript. SG, LN, AG-M, JL-E, and MH contributed to critical revising and editing the manuscript. All authors read and approved final manuscript.

## Conflict of Interest

The authors declare that the research was conducted in the absence of any commercial or financial relationships that could be construed as a potential conflict of interest.

## Supplementary Material

Supplementary Figure 1 Flow chart depicting systematic review of genes implicated in Meniere's disease. Results of PRISMA search strategy and PubTerm search were combined and PRISMA systematic review criteria were applied to all identified references. In total, 389 unique abstracts were identified. Abstracts unrelated to Meniere's disease (*n* = 196) were excluded. The following exclusion criteria including non-English language (*n* = 22), animal studies (*n* = 21), non-gene outcome defined as an absence of genes studied in relation to Meniere's disease (*n* = 45), and unrelated to Meniere's disease (*n* = 28) were applied to a full-text review of these references resulting in 77 references being included for systematic review.

Supplementary Figures 2–4 Expression of Meniere's disease implicated genes without cell type-specific expression in the adult mouse SV as demonstrated by single-cell RNA-Seq. Heatmap displays cell types along the horizontal axis and genes along vertical axis. Gene expression is displayed in normalized counts. Cell types displayed include marginal cells, intermediate cells, basal cells, spindle-root cells, fibrocytes, and macrophages.

Supplementary Figures 5–9 Expression of Meniere's disease implicated genes without cell type-specific expression in the adult mouse SV as demonstrated by single-nucleus RNA-Seq. Heatmap displays cell types along the horizontal axis and genes along vertical axis. Gene expression is displayed in normalized counts. Cell types displayed include marginal cells, intermediate cells, basal cells, spindle cells, root cells, Reissner's membrane cells, and macrophages.

Supplementary Figure 10 Expression of Meniere's disease implicated genes in the developing mouse cochlea as see in the Allen Brain Atlas. **(A)** In the E15.5 mouse, *Sgk1* is expressed in the organ of Cort and the roof of the cochlear duct where future marginal cells reside. **(B)** In the E15.5 mouse, Cacna2d1 is localized to the roof of the cochlear duct where future marginal cells reside. **(C)** Eya4 is widely expressed in the cochlear duct including the region of the future stria vascularis at E15.5. Scale bars are 200 microns.

Supplementary Data 1 Descriptive tables summarizing studies in humans implicating genes in Meniere's disease.

Supplementary Data 2 Accompanying lists of genes for human studies that examined larger groups of genes as referred to in Supplementary Data 1.

Supplementary Table 1 Genes investigated in relation to Meniere's disease.
