# Supplementary material for: Identification of Potential Meniere's Disease Targets in the Adult Stria Vascularis
Source: Front Neurol. 2021 Feb 5;12:630561. doi: 10.3389/fneur.2021.630561 (PMC7894210; doi:10.3389/fneur.2021.630561)
Supplement: Supplementary Figure 1 — Flow chart depicting systematic review of genes implicated in Meniere's disease. Results of PRISMA search strategy and PubTerm search were combined and PRISMA systematic review criteria were applied to all identified references. In total, 389 unique abstracts were identified. Abstracts unrelated to Meniere's disease (n = 196) were excluded. The following exclusion criteria including non-English language (n = 22), animal studies (n = 21), non-gene outcome defined as an absence of genes studied in relation to Meniere's disease (n = 45), and unrelated to Meniere's disease (n = 28) were applied to a full-text review of these references resulting in 77 references being included for systematic review. [file Image_1.PDF]

PRISMA Flow Diagram: Meniere's Genes Systematic Review

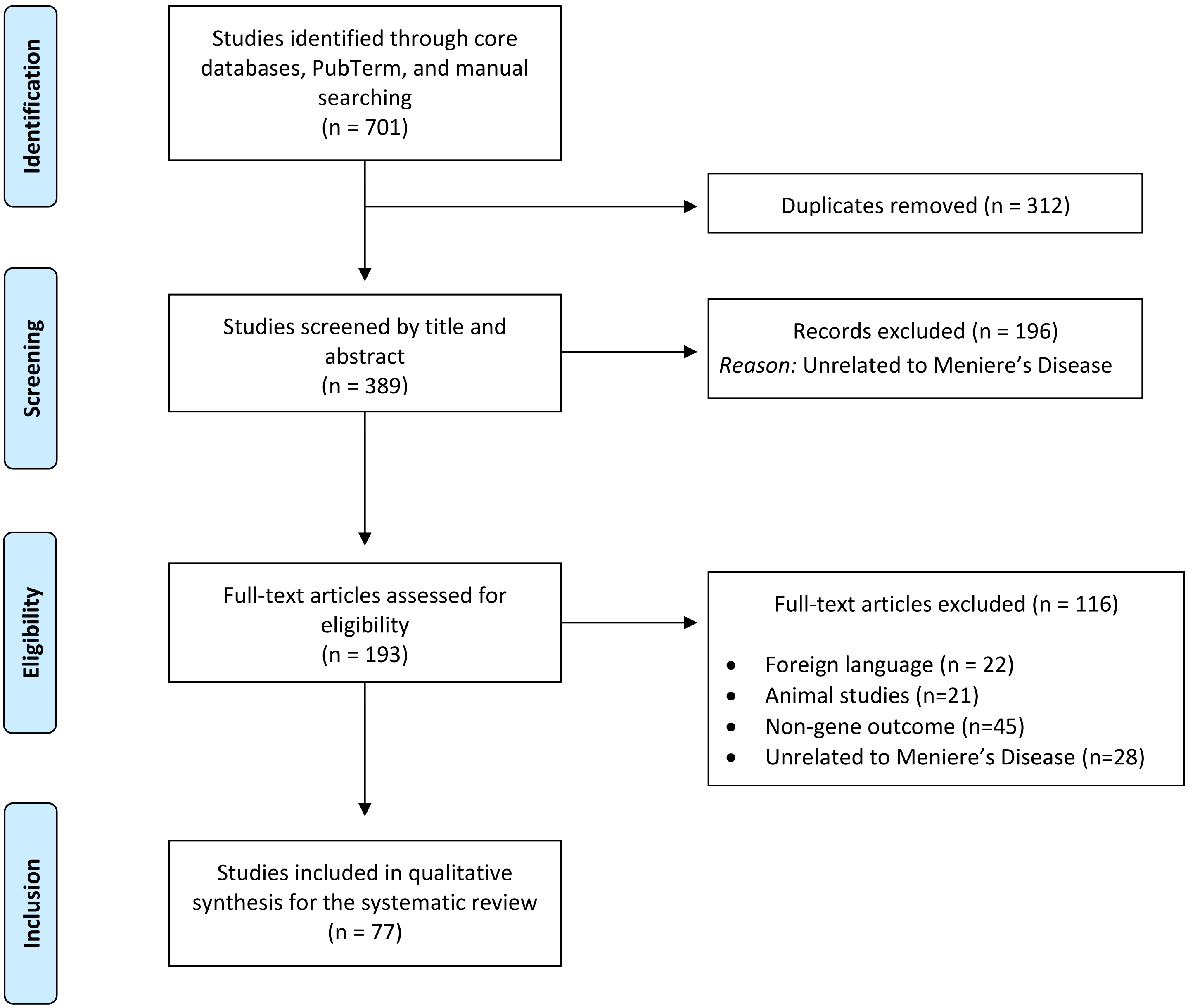

From: Moher D, Liberati A, Tetzlaff J, Altman DG, The PRISMA Group (2009). *P*referred *R*eporting *I*tems for *S*ystematic Reviews and *M*eta-*A*nalyses: The PRISMA Statement. PLoS Med 6(7): e1000097. doi:10.1371/journal.pmed1000097

For more information, visit www.prisma-statement.org.
