# Supplementary figures and images for: Identification of Potential Meniere's Disease Targets in the Adult Stria Vascularis
Source: Front Neurol. 2021 Feb 5;12:630561. doi: 10.3389/fneur.2021.630561 (PMC7894210; doi:10.3389/fneur.2021.630561)

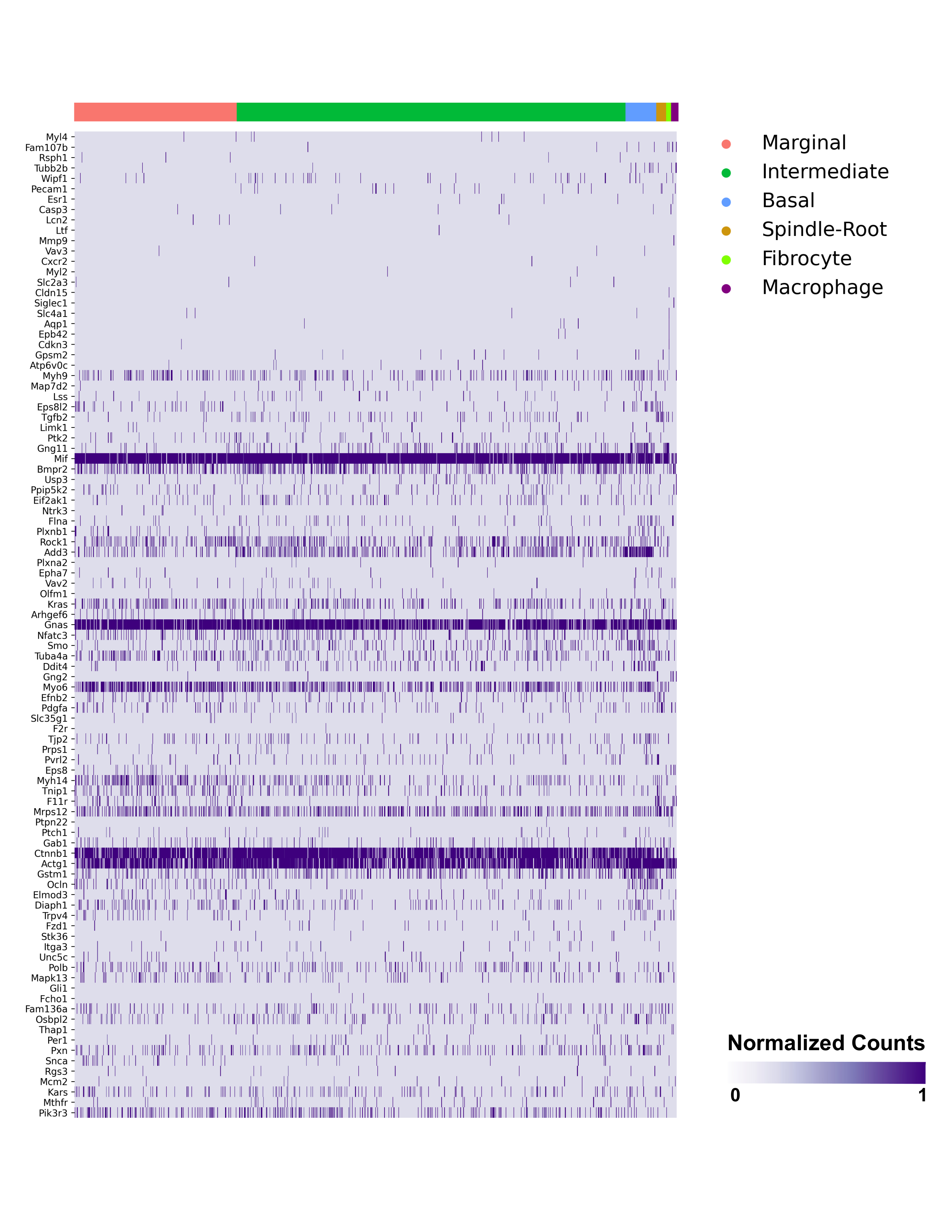

Supplement: Supplementary file 2 [file Image_2.JPEG]

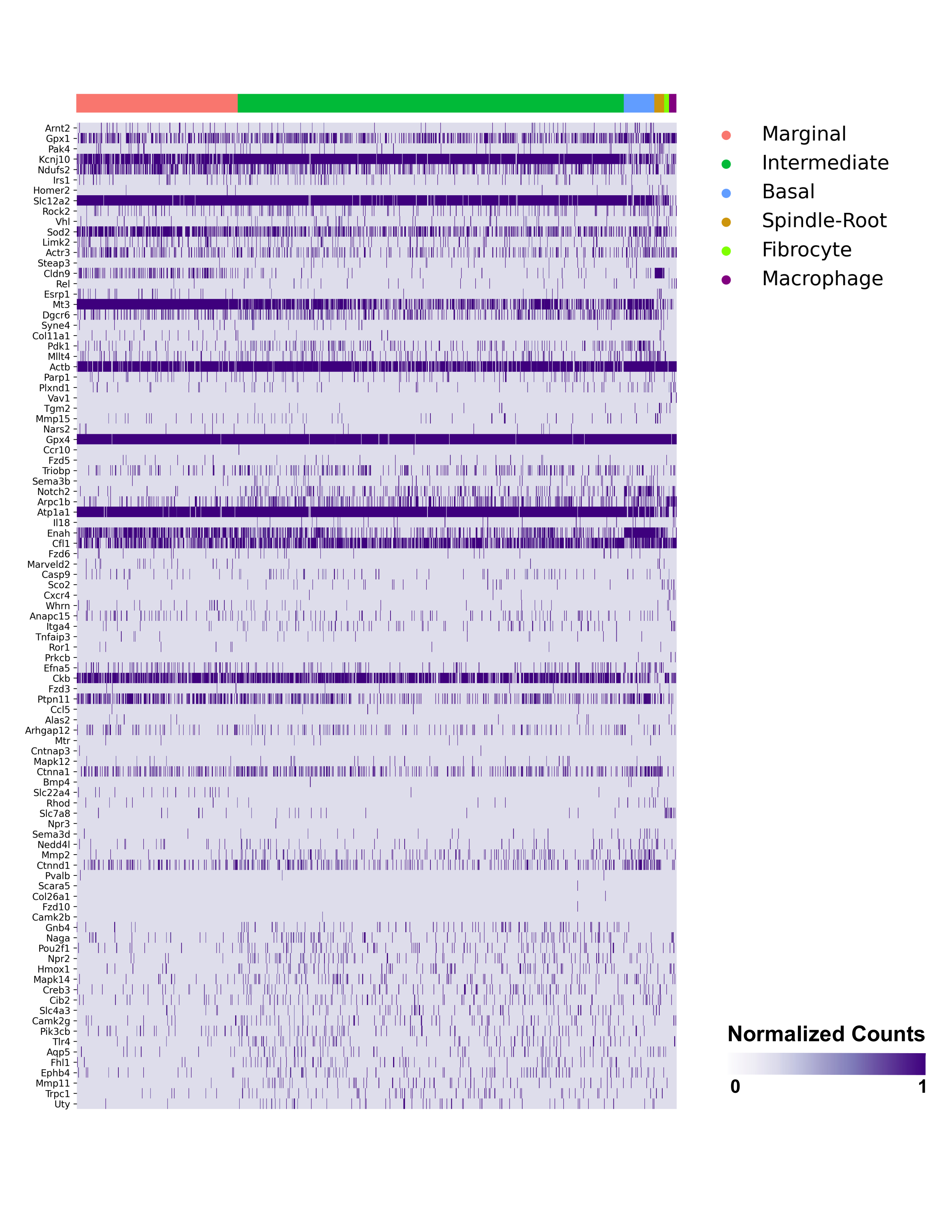

Supplement: Supplementary file 3 [file Image_3.JPEG]

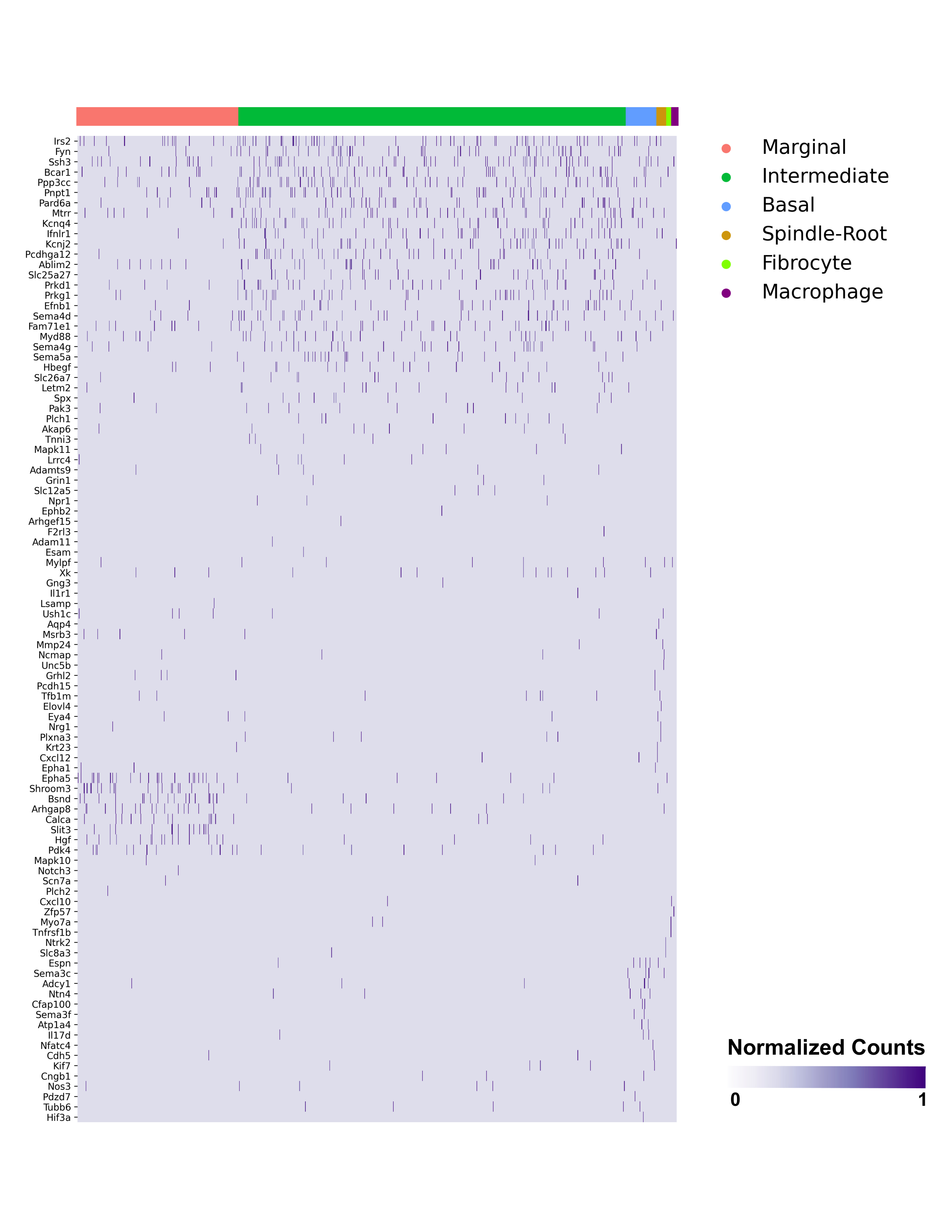

Supplement: Supplementary Figures 2–4 — Expression of Meniere's disease implicated genes without cell type-specific expression in the adult mouse SV as demonstrated by single-cell RNA-Seq. Heatmap displays cell types along the horizontal axis and genes along vertical axis. Gene expression is displayed in normalized counts. Cell types displayed include marginal cells, intermediate cells, basal cells, spindle-root cells, fibrocytes, and macrophages. [file Image_4.JPEG]

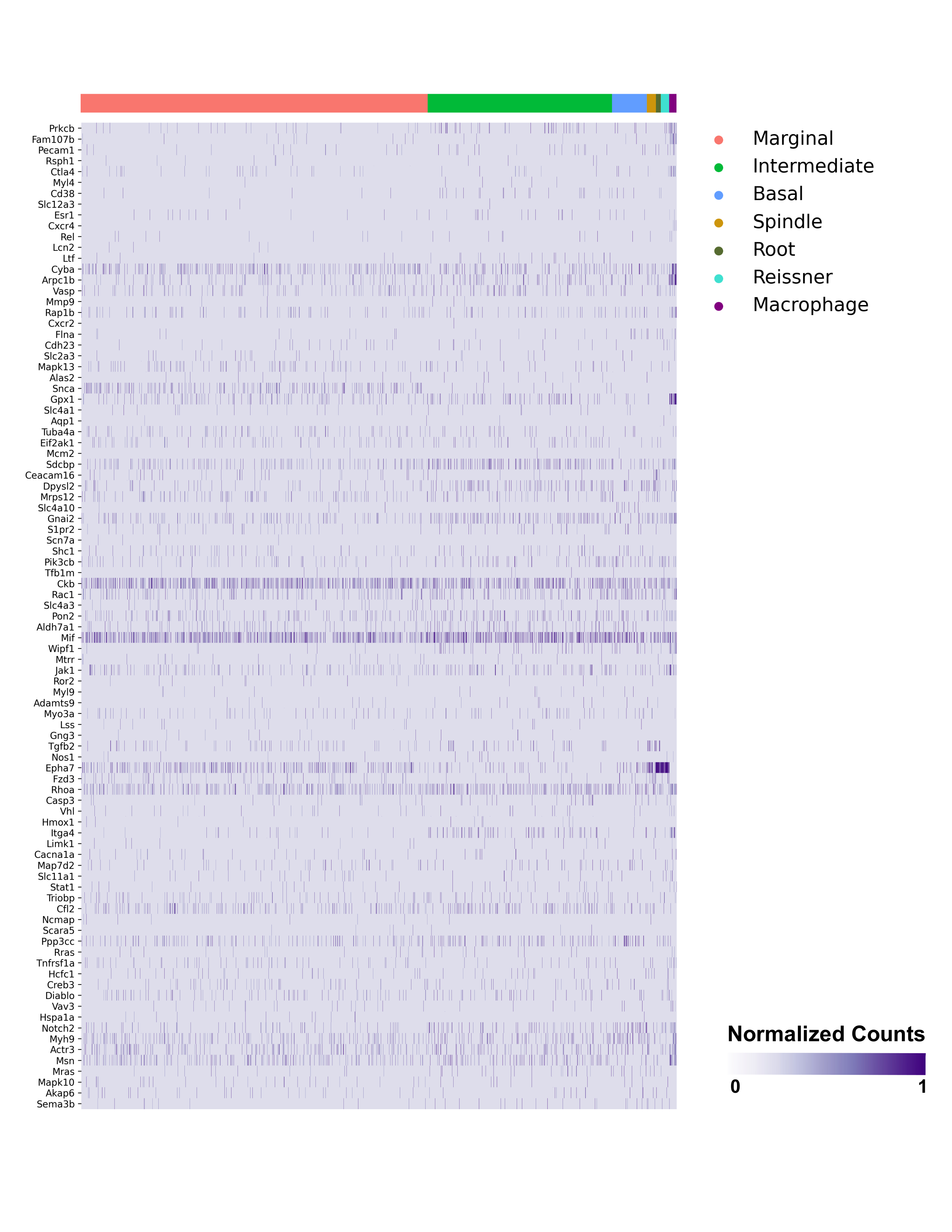

Supplement: Supplementary file 5 [file Image_5.JPEG]

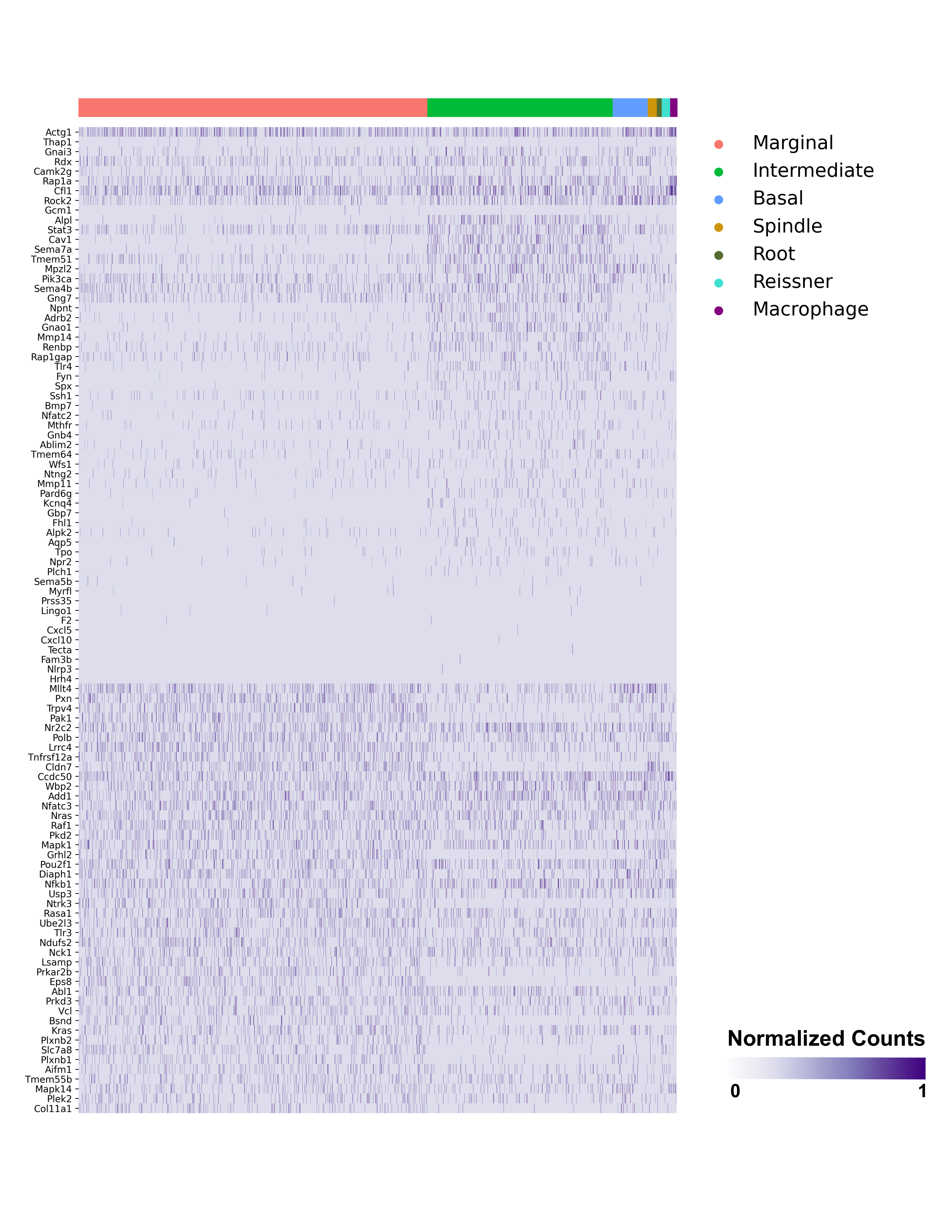

Supplement: Supplementary file 6 [file Image_6.JPEG]

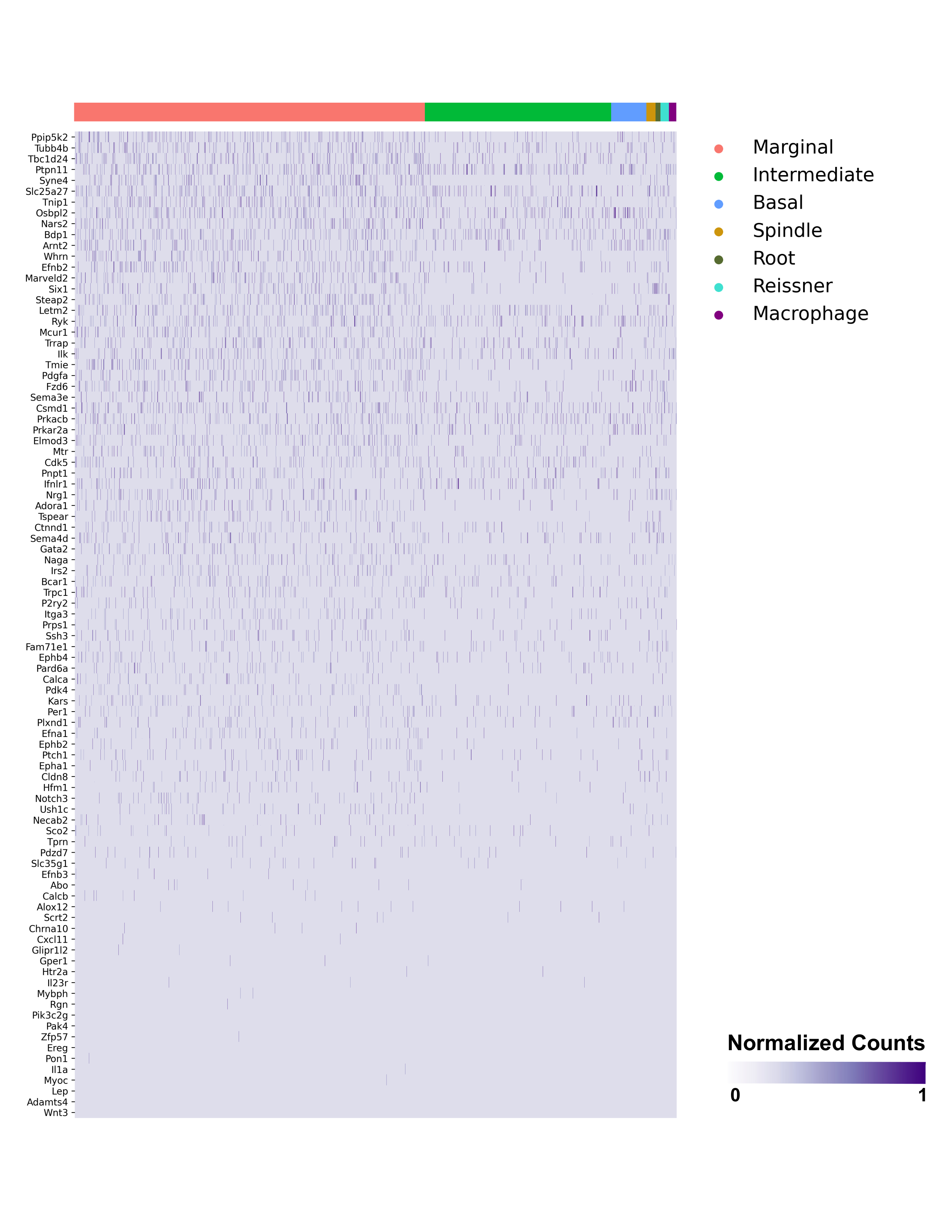

Supplement: Supplementary file 7 [file Image_7.JPEG]

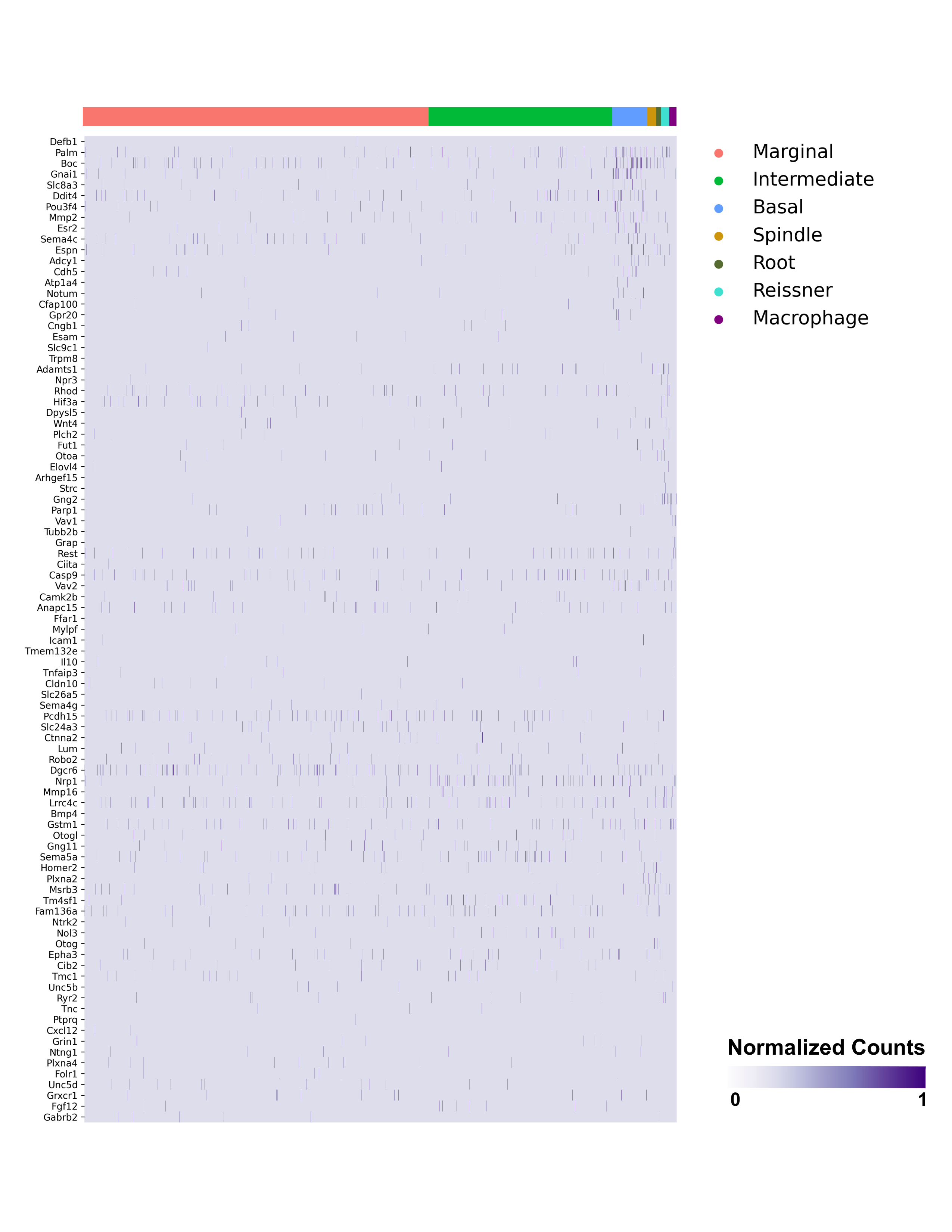

Supplement: Supplementary file 8 [file Image_8.JPEG]

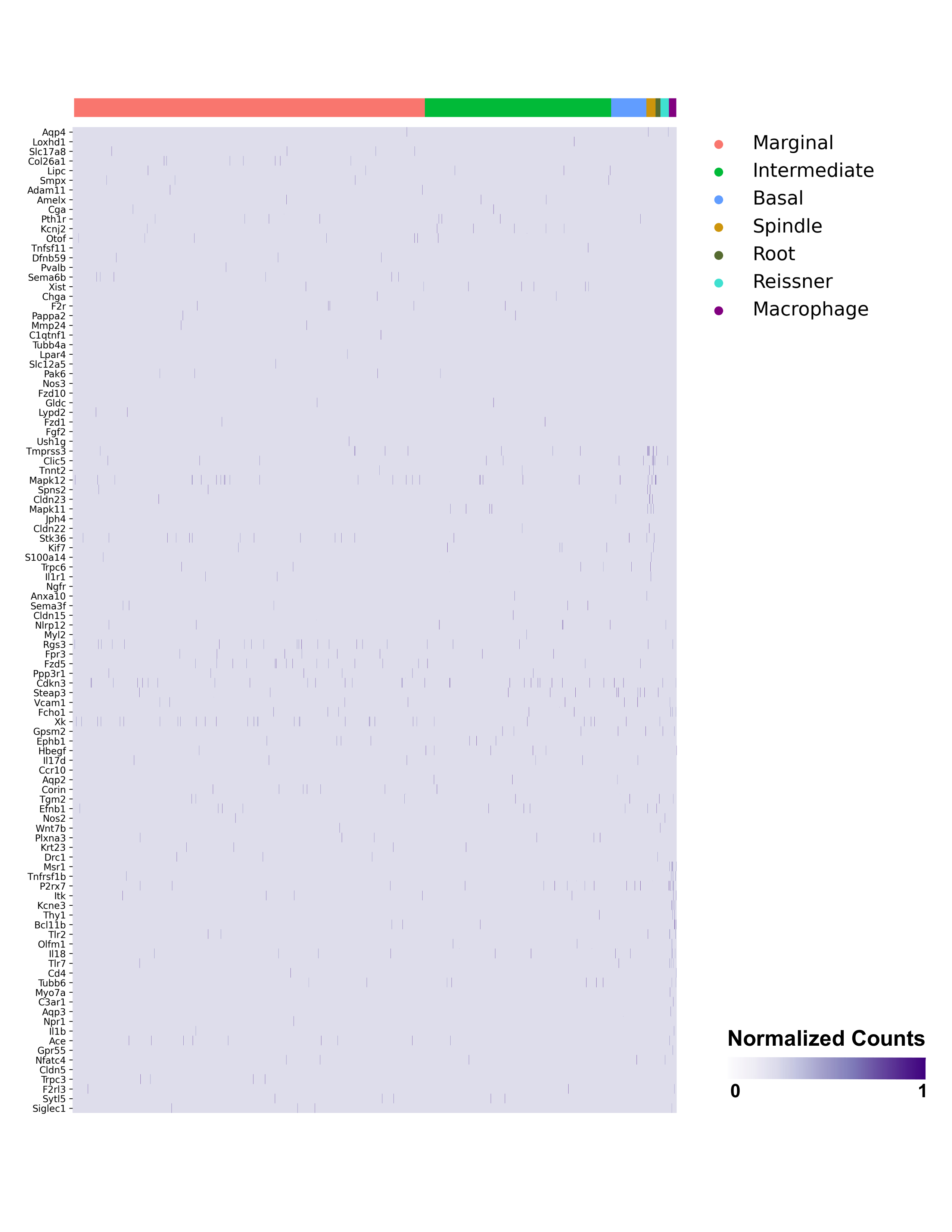

Supplement: Supplementary Figures 5–9 — Expression of Meniere's disease implicated genes without cell type-specific expression in the adult mouse SV as demonstrated by single-nucleus RNA-Seq. Heatmap displays cell types along the horizontal axis and genes along vertical axis. Gene expression is displayed in normalized counts. Cell types displayed include marginal cells, intermediate cells, basal cells, spindle cells, root cells, Reissner's membrane cells, and macrophages. [file Image_9.JPEG]

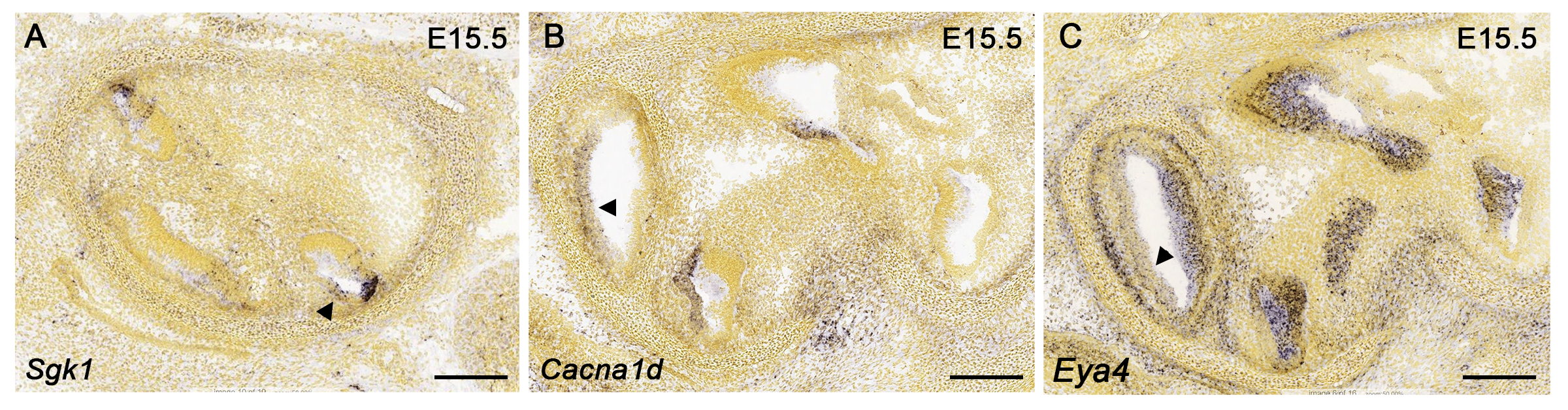

Supplement: Supplementary Figure 10 — Expression of Meniere's disease implicated genes in the developing mouse cochlea as see in the Allen Brain Atlas. (A) In the E15.5 mouse, Sgk1 is expressed in the organ of Cort and the roof of the cochlear duct where future marginal cells reside. (B) In the E15.5 mouse, Cacna2d1 is localized to the roof of the cochlear duct where future marginal cells reside. (C) Eya4 is widely expressed in the cochlear duct including the region of the future stria vascularis at E15.5. Scale bars are 200 microns. [file Image_10.TIF]
