# Supplementary material for: Identification of Potential Meniere's Disease Targets in the Adult Stria Vascularis
Source: Front Neurol. 2021 Feb 5;12:630561. doi: 10.3389/fneur.2021.630561 (PMC7894210; doi:10.3389/fneur.2021.630561)
Supplement: Supplementary Data 1 — Descriptive tables summarizing studies in humans implicating genes in Meniere's disease. [file Data_Sheet_1.PDF]

Supplemental Data S1. Summary of Studies Investigating Gene Involvement in Meniere's Disease

First Author	Year	Study Design	Population	SMD/FMD	# MD Subjects	Males	Females	Mean Age ± SD	Investigated Genes
Arweiler-Harbeck D	2012	Case control	German	Both	102	40	62	59.1±14.3	***AQP5***
Asmar MH	2018	Case control	Canadian	Sporadic	24	6	18	54.3	***AQP2, AVPR2, SLC12A1, TRPV4***
Bernstein JM	1996	Case control	NR	Sporadic	52	NR	NR	NR	***MHC DQw2-DR3-C4BSf-C4A0-G11:15-Bf:0.4-C2a-HSP70:7.5-TNF a5-B8-Cw7-A1***
Cabrera S	2014	Case control	Spanish	Sporadic	716	210	506	46.75 ± 17.4	***NFKB1, REL, TNFAIP3, TNIP1, UBE2L3***
Campbell CA	2010	Case control	Caucasian	Sporadic	180	78	102	54.3	***KCNE1, KCNE3***
Candreia C	2010	Case control	Swiss	Sporadic	34	18	16	45	***AQP1, AQP2, AQP3, AQP4***
Chan KC	2018	Case control	Taiwanese	Sporadic	35	14	21	53.9 ± 13.6	***HLA-A, HLA-B, HLA-C, HLA-DQ***
Dai Q	2019	Case control	Chinese	Both	24	8	16	37 ± 20.6	***KCNE1, KCNE3***
Doi K	2005	Case control	Japanese	Sporadic	63	18	45	42.5	***KCNE1, KCNE3***
Fransen E	1999	Case study	Belgian/Dutch	Familial	46	23	23	NR	***COCH***
Frejo L	2017	Case control	Spanish	Sporadic	1451	609	842	47.24 ± 12.1	***NFKB1, TNF, TNFRSF12A, TNFSF12***
Frykholm C	2006	Case study	Swedish	Familial	12	5	7	44.2	***COCH, DIAPH1, POU4F3, WFS1***
Furuta T	2011	Case control	Japanese	Sporadic	68	28	40	56.3 ± 13.4	***IL1A, IL1B***
Gabrikova D	2010	Case study	Swedish	Familial	24	NR	NR	NR	***PIK3C2G, RERGL***
Gallego-Martinez A	2020	Cohort	Iberian	Sporadic	920	NR	NR	NR	**263 genes; See Supplemental Data S2**
Gallego-Martinez A	2019	Cohort	Iberian	Sporadic	890	356	534	NR	**45 genes; See Supplemental Data S2**
Gazquez I	2011	Case control	Spanish; European American	Sporadic	386	179	207	55.55 ± 18.9	***NOS1, NOS2***

Gazquez I	2013	Case control	Spanish; European American	Sporadic	580	270	310	56.35 ± 17.4	***IFNG, MIF, TNF***
Gazquez I	2012	Case control	Spanish	Sporadic	302	142	160	55.15 ± 18.7	***HLA-B, MICA***
Hietikko E	2012	Case study	Finnish	Both	95	NR	NR	NR	***ADD1, AQP2, COCH, HCFC1, KCNE1, KCNE3***
Hietikko E	2011	Case study	Finnish	Familial	38	24	14	21 – 62	**Chromosome 12p12.13**
Huang CJ	2019	Cohort	Taiwanese	Sporadic	21	11	10	57.3 ± 10.9	***ALPL, AOC1, CASP9, CIITA, CYP1A1, CYP2B6***
Huang Y	2013	Case control	Japanese	Sporadic	86	32	54	49.506 ± 15.0555	***MTHFR***
Ishiyama G	2006	Review	N/A	N/A	N/A	N/A	N/A	N/A	***AQP1, AQP2, AQP3, AQP4, AQP5, AQP6***
Ishiyama G	2010	Case control	NR	Sporadic	6	4	2	76	***AQP1, AQP4, AQP6***
Kawaguchi S	2008	Case control	Japanese	Sporadic	49	20	29	32.9	***HSPA1A***
Khorsandi MT	2011	Case control	Iranian	Sporadic	21	9	12	NR	***HLA-C***
Kim BG	2017	Case control	Korean	Sporadic	10	5	5	13.6	***ADRB1, ADRB2***
Kitahara T	2009	Case control	Japanese	Sporadic	87	38	49	47.2 ± 1.4	***AVPR2***
Klar J	2006	Case study	Swedish	Familial	20	6	14	NR	***PIK3C2G***
Koo JW	2003	Case control	Korean	Sporadic	41	16	25	47.4	***HLA-DRB1***
Koyama S	1993	Case control	Japanese	Sporadic	20	9	11	54.9 ± 12.6	***HLA-A, HLA-B, HLA-C, HLA-DQ, HLA-DR***
Kumagami H	2009	Cohort	Japanese	Sporadic	6	NR	NR	39.3	***TRPV4***
Lemaire FX	2003	Case study	Belgian	Familial	NR	NR	NR	NR	***COCH***
Li L	2017	Genetic inference study	N/A	Both	N/A	N/A	N/A	N/A	**84 genes; See Supplemental Data S2**
Lopes KC	2016	Case control	Caucasian	Sporadic	30	19	11	41.5	***AQP2, AQP3, KCNE1***
Lopez-Escamez JA	2010	Case control	Spanish	Sporadic	52	20	32	57 ± 10	***CTLA4, PTPN22***

Lopez-Escamez JA	2002	Case control	Spanish	Sporadic	54	28	26	51.05 ± 11.19	***HLA-DRB1***
Lopez-Escamez JA	2007	Case control	Spanish	Sporadic	80	38	42	56.6 ± 15.5	***HLA-DRB1, HLA-DQB1***
Lopez-Escamez JA	2009	Case control	Spanish	Sporadic	80	38	42	56.6 ± 15.5	***PARP1***
Lopez-Escamez JA	2011	Case control	Spanish	Sporadic	268	128	140	55.8 ± 19.0	***CD16A, CD32A***
Lopez-Escamez JA	2018	Review	N/A	Both	N/A	N/A	N/A	N/A	***COCH, DPT, DTNA, FAM136A, IL1B, IL6, NFKB1, PRKCB, SEMA3D, TNF***
Lynch M	2002	Case study	NR	Familial	13	6	7	15 – 62	***ALDH7A1***
Maekawa C	2010	Case control	Japanese	Sporadic	15	7	8	48.6 ± 5.8	***AQP2***
Mallur P.S.	2010	Case control	Northern European	Sporadic	30	13	17	64 ± 12.7	***AQP1, AQP2, AQP3, AQP4***
Martin-Sierra C	2016	Case study	Spanish	Familial	2	2	-	48	***PRKCB***
Martin-Sierra C	2017	Case study	Spanish	Familial	6	2	4	NR	***DPT, SEMA3D***
Mehrjoo Z	2020	Case study	Iranian	Familial	2	-	2	37.5	***BAGE, CFAP100, KMT2C, LSAMP, OR5H2, OR5K4, USP3, ZBED2***
Melchiorri L	2002	Case control	Italian	Sporadic	41	NR	NR	NR	***HLA-A, HLA-B, HLA-C, HLA-DR***
Mhatre AN	2002	Cohort	NR	Sporadic	12	NR	NR	NR	***AQP2***
Mohseni M	2020	Case control	Iranian	Sporadic	64	20	44	38.07 ± 11.53	***ACE***
Morrison AW	1994	Cohort	NR	Both	97	NR	NR	NR	***HLA-A, HLA-B, HLA-C***
Nair TS	2016	Case control	American	Sporadic	52	NR	NR	NR	***SLC44A2***
Nishio N	2013	Case control	Japanese	Sporadic	86	32	54	54.4 ± 15.3	***AQP4, AQP5, ESR1***
Oh EH	2020	Case control	Korean	Sporadic	68	38	30	60.2 ± 12.0	**45 genes; See Supplemental Data S2**
Pacheu-Grau D	2012	Case control	Spanish	Sporadic	24	9	15	53.5 ± 13.7	***TFB1M, MT-RNR1, MT-RNR2, MRPS12***

Qin D	2019	Case control	Chinese	Sporadic	90	42	48	62 ± 12.8	***HRH4***
Rawal SG	2010	Case study	American	Sporadic	2	2	-	67.5	***HLA-B***
Requena T	2019	Case study	Spanish	Familial	NR	NR	NR	NR	***NLRP12***
Requena T	2013	Case control	Spanish, Italian	Sporadic	1013	527	486	55.7 ± 12.5	***TLR3, TLR7, TLR8, TLR10***
Requena T	2015	Case study	Spanish	Familial	3	-	3	NR	***DTNA, FAM136A***
Roman-Naranjo P	2020	Cohort	Spanish	Both	82	25	57	33.7	**116 genes; See Supplemental Data S2**
Sanchez E	2004	Case control	Spanish	Sporadic	30	12	18	54	***COCH***
Skarp S	2019	Case study	Finnish	Familial	2	2	-	11, NR	***HMX2, TMEM55B***
Sun Y	2018	Cohort	Chinese	Sporadic	3	NR	NR	NR	**366 genes; See Supplemental Data S2**
Teggi R	2017	Case control	Caucasian	Sporadic	155	69	86	53.7 ± 11.6	**20 genes; See Supplemental Data S2**
Teggi R	2008	Case control	Caucasian	Sporadic	28	11	17	46 ± 13.8	***ADD1, ADD2, ADD3***
Teranishi M	2012	Case control	Japanese	Sporadic	84	38	46	58.2 ± 14.3	***GPX1, PON1, PON2, SOD2***
Teranishi M	2013	Case control	Japanese	Sporadic	83	31	52	54.9 ± 15.3	***CAV1, CYBA, MT5178. MTNR1B, MTR, MTRR, NOS3***
Tsukada K	2020	Case study	Japanese	Sporadic	5	-	5	25.2	***SLC26A4***
Usami S	2003	Case study	Japanese	Sporadic	20	NR	NR	50.15	***COCH***
Verhagen WI	2001	Case study	Dutch	Familial	9	4	5	51.2	***COCH***
Vrabec JT	2008	Case control	European American	Sporadic	30	NR	NR	NR	**40 genes; See Supplemental Data S2**
Yazdani N	2015	Case control	Iranian	Sporadic	71	25	46	39.7 ± 11.5	***CCL5***
Yazdani N	2013	Case control	Iranian	Sporadic	72	25	47	37.7 ± 11.3	***MIF***
Yeo SW	2002	Case control	Korean	Sporadic	39	20	19	50.2 ± 11.4	***HLA-A, HLA-B, HLA-C, HLA-DRB1, HLA-DQA1, HLA-DQB1***
Yoshida T	2015	Case study	Japanese	Sporadic	1	-	1	13	***SLC26A4***
