# Supplementary material for: Identification of Potential Meniere's Disease Targets in the Adult Stria Vascularis
Source: Front Neurol. 2021 Feb 5;12:630561. doi: 10.3389/fneur.2021.630561 (PMC7894210; doi:10.3389/fneur.2021.630561)
Supplement: Supplementary Table 1 — Genes investigated in relation to Meniere's disease. [file Table_1.docx]

**Supplemental Tables**

**Supplemental Table S1.** Genes Investigated in Relation to Meniere’s Disease

| **Gene** | **# Total Studies** | **Supporting Studies** | **Non-Supporting Studies** |
| --- | --- | --- | --- |
| ***AADAC*** | 1 | Sun 2018 | - |
| ***ABO*** | 1 | Sun 2018 | - |
| ***ABL1*** | 1 | - | Gallego-Martinez 2020 |
| ***ABLIM1*** | 1 | - | Gallego-Martinez 2020 |
| ***ABLIM2*** | 1 | - | Gallego-Martinez 2020 |
| ***ACE*** | 1 | Mohseni 2020 | - |
| ***ACTB*** | 1 | - | Gallego-Martinez 2020 |
| ***ACTC1*** | 1 | - | Gallego-Martinez 2020 |
| ***ACTG1*** | 2 | Roman-Naranjo 2020 | Gallego-Martinez 2019 |
| ***ACTN1*** | 1 | Gallego-Martinez 2020 | - |
| ***ACTN4*** | 1 | - | Gallego-Martinez 2020 |
| ***ACTR3*** | 1 | - | Gallego-Martinez 2020 |
| ***ADAM11*** | 1 | - | Gallego-Martinez 2020 |
| ***ADAM23*** | 1 | - | Gallego-Martinez 2020 |
| ***ADAMTS1*** | 1 | Gallego-Martinez 2020 | - |
| ***ADAMTS4*** | 1 | Gallego-Martinez 2020 | - |
| ***ADAMTS9*** | 1 | Gallego-Martinez 2020 | - |
| ***ADCY1*** | 1 | Roman-Naranjo 2020 | - |
| ***ADD1*** | 6 | Teggi 2008, Li 2017, Gallego-Martinez 2019 | Hietikko 2012, Teggi 2017, Oh 2020 |
| ***ADD2*** | 3 | Li 2017 | Teggi 2008, Teggi 2017 |
| ***ADD3*** | 3 | Li 2017 | Teggi 2008, Teggi 2017 |
| ***ADORA1*** | 1 | Sun 2018 | - |
| ***ADRB1*** | 1 | Kim 2017 | - |
| ***ADRB2*** | 1 | Kim 2017 | - |
| ***AIFM1*** | 1 | Roman-Naranjo 2020 | - |
| ***AKAP6*** | 1 | Sun 2018 | - |
| ***ALAS2*** | 1 | Sun 2018 | - |
| ***ALDH7A1*** | 2 | Roman-Naranjo 2020 | Lynch 2002 |
| ***ALOX12*** | 1 | Sun 2018 | - |
| ***ALPK2*** | 1 | Sun 2018 | - |
| ***ALPL*** | 2 | Sun 2018 | Huang 2019 |
| ***ALPL1*** | 1 | Sun 2018 | - |
| ***AMELX*** | 1 | Sun 2018 | - |
| ***ANAPC15*** | 1 | Roman-Naranjo 2020 | - |
| ***ANK2*** | 1 | Sun 2018 | - |
| ***ANXA10*** | 1 | Sun 2018 | - |
| ***AOC1*** | 2 | Sun 2018 | Huang 2019 |
| ***AQP1*** | 5 | Ishiyama 2006, Li 2017 | Candreia 2010, Ishiyama 2010, Mallur 2010 |
| ***AQP2*** | 9 | Ishiyama 2006, Maekawa 2010, Lopes 2016, Li 2017, Asmar 2018 | Mhatre 2002, Candreia 2010, Mallur 2010, Hietikko 2012 |
| ***AQP3*** | 5 | Ishiyama 2006, Candreia 2010, Mallur 2010, Lopes 2016, Li 2017 | - |
| ***AQP4*** | 7 | Ishiyama 2006, Li 2017 | Candreia 2010, Ishiyama 2010, Mallur 2010, Nishio 2013, Oh 2020 |
| ***AQP5*** | 4 | Nishio 2013, Li 2017 | Arweiler-Harbeck 2012, Oh 2020 |
| ***AQP6*** | 2 | Li 2017 | Ishiyama 2010 |
| ***AREG*** | 1 | Sun 2018 | - |
| ***ARHGAP12*** | 1 | - | Gallego-Martinez 2020 |
| ***ARHGAP5*** | 1 | Gallego-Martinez 2020 | - |
| ***ARHGAP8*** | 1 | Gallego-Martinez 2020 | - |
| ***ARHGEF15*** | 1 | - | Gallego-Martinez 2020 |
| ***ARHGEF6*** | 1 | - | Gallego-Martinez 2020 |
| ***ARNT2*** | 1 | - | Gallego-Martinez 2019 |
| ***ARPC1B*** | 1 | Gallego-Martinez 2020 | - |
| ***ATP13A5*** | 1 | Sun 2018 | - |
| ***ATP1A1*** | 1 | - | Vrabec 2008 |
| ***ATP1A2*** | 1 | - | Vrabec 2008 |
| ***ATP1A4*** | 1 | Sun 2018 | - |
| ***ATP1B2*** | 1 | Sun 2018 | Vrabec 2008 |
| ***ATP6V0C*** | 1 | Sun 2018 | - |
| ***ATP6V1G3*** | 1 | Sun 2018 | - |
| ***AVPR2*** | 3 | Kitahara 2009, Li 2017 | Asmar 2018 |
| ***BAGE*** | 1 | - | Mehrjoo 2020 |
| ***BCAR1*** | 1 | - | Gallego-Martinez 2020 |
| ***BCL11B*** | 1 | - | Vrabec 2008 |
| ***BDP1*** | 1 | Roman-Naranjo 2020 | - |
| ***BDNF*** | 1 | - | Gallego-Martinez 2020 |
| ***BMP1*** | 1 | Gallego-Martinez 2020 | - |
| ***BMP4*** | 1 | - | Gallego-Martinez 2020 |
| ***BMP6*** | 2 | Sun 2018, Gallego-Martinez 2020 | - |
| ***BMP7*** | 1 | - | Gallego-Martinez 2020 |
| ***BMPR1B*** | 1 | - | Gallego-Martinez 2020 |
| ***BMPR2*** | 1 | - | Gallego-Martinez 2020 |
| ***BOC*** | 1 | - | Gallego-Martinez 2020 |
| ***BSND*** | 1 | Roman-Naranjo 2020 | - |
| ***BTBD17*** | 1 | Sun 2018 | - |
| ***BTNL3*** | 1 | Sun 2018 | - |
| ***BTNLB*** | 1 | Sun 2018 | - |
| ***C10orf105*** | 1 | Roman-Naranjo 2020 | - |
| ***C1QA*** | 1 | Sun 2018 | - |
| ***C1QB*** | 1 | Sun 2018 | - |
| ***C1QTNF1*** | 1 | Sun 2018 | - |
| ***C3AR1*** | 1 | Sun 2018 | - |
| ***CA2*** | 1 | Sun 2018 | - |
| ***CABP2*** | 1 | Roman-Naranjo 2020 | - |
| ***CACNA1A*** | 3 | Li 2017, Sun 2018 | Vrabec 2008 |
| ***CACNA2D1*** | 1 | Sun 2018 | - |
| ***CALCA*** | 1 | Sun 2018 | - |
| ***CALCB*** | 1 | Sun 2018 | - |
| ***CAMK2B*** | 1 | - | Gallego-Martinez 2020 |
| ***CAMK2D*** | 1 | - | Gallego-Martinez 2020 |
| ***CAMK2G*** | 1 | - | Gallego-Martinez 2020 |
| ***CAR12*** | 1 | Sun 2018 | - |
| ***CASP3*** | 1 | Vrabec 2008 | - |
| ***CASP9*** | 1 | Huang 2019 | - |
| ***CAV1*** | 4 | Teranishi 2013, Li 2017, Sun 2018 | Oh 2020 |
| ***CCDC50*** | 1 | Gallego-Martinez 2019 | - |
| ***CCKAR*** | 1 | Sun 2018 | - |
| ***CCL14*** | 1 | Sun 2018 | - |
| ***CCL15*** | 1 | Sun 2018 | - |
| ***CCL16*** | 1 | Sun 2018 | - |
| ***CCL2*** | 1 | - | Oh 2020 |
| ***CCL23*** | 1 | Sun 2018 | - |
| ***CCL3L3*** | 1 | Sun 2018 | - |
| ***CCL4L2*** | 1 | Sun 2018 | - |
| ***CCL5*** | 3 | Yazdani 2015, Li 2017 | Vrabec 2008, Oh 2020 |
| ***CCL8*** | 1 | Sun 2018 | - |
| ***CCR10*** | 1 | Sun 2018 | - |
| ***CCR3*** | 1 | Sun 2018 | - |
| ***CD16A*** | 1 | - | Lopez-Escamez 2011 |
| ***CD32A*** | 1 | - | Lopez-Escamez 2011 |
| ***CD38*** | 1 | Sun 2018 | - |
| ***CD4*** | 1 | - | Oh 2020 |
| ***CDC14A*** | 1 | Roman-Naranjo 2020 | - |
| ***CDC42*** | 1 | - | Gallego-Martinez 2020 |
| ***CDH5*** | 1 | - | Gallego-Martinez 2020 |
| ***CDH23*** | 1 | Roman-Naranjo 2020 | - |
| ***CDHR4*** | 1 | Sun 2018 | - |
| ***CDK5*** | 1 | - | Gallego-Martinez 2020 |
| ***CDKN3*** | 1 | - | Vrabec 2008 |
| ***CEACAM16*** | 1 | Gallego-Martinez 2019 | - |
| ***CEACAM22P*** | 1 | Sun 2018 | - |
| ***CFAP100*** | 1 | - | Mehrjoo 2020 |
| ***CFL1*** | 1 | - | Gallego-Martinez 2020 |
| ***CFL2*** | 1 | - | Gallego-Martinez 2020 |
| ***CGA*** | 1 | Li 2017 | - |
| ***CHGA*** | 2 | Li 2017 | Oh 2020 |
| ***CHI3L1*** | 1 | Sun 2018 | - |
| ***CHRM4*** | 1 | Sun 2018 | - |
| ***CHRNA10*** | 1 | Sun 2018 | - |
| ***CIB2*** | 1 | Roman-Naranjo 2020 | - |
| ***CIITA*** | 1 | - | Huang 2019 |
| ***CIR2DP1*** | 1 | Sun 2018 | - |
| ***CKB*** | 1 | Sun 2018 | - |
| ***CLDN10*** | 1 | - | Gallego-Martinez 2020 |
| ***CLDN11*** | 1 | - | Gallego-Martinez 2020 |
| ***CLDN14*** | 2 | Gallego-Martinez 2019, Roman-Naranjo 2020 | - |
| ***CLDN15*** | 1 | - | Gallego-Martinez 2020 |
| ***CLDN16*** | 2 | Sun 2018 | Gallego-Martinez 2020 |
| ***CLDN22*** | 1 | - | Gallego-Martinez 2020 |
| ***CLDN23*** | 1 | Sun 2018 | - |
| ***CLDN3*** | 1 | - | Gallego-Martinez 2020 |
| ***CLDN5*** | 1 | - | Gallego-Martinez 2020 |
| ***CLDN6*** | 1 | - | Gallego-Martinez 2020 |
| ***CLDN7*** | 1 | Gallego-Martinez 2020 | - |
| ***CLDN8*** | 1 | Gallego-Martinez 2020 | - |
| ***CLDN9*** | 2 | Roman-Naranjo 2020 | Gallego-Martinez 2020 |
| ***CLIC2*** | 1 | Sun 2018 | - |
| ***CLIC5*** | 1 | Roman-Naranjo 2020 | - |
| ***CLLU1OS*** | 1 | Sun 2018 | - |
| ***CNGB1*** | 1 | Sun 2018 | - |
| ***CNTF*** | 1 | - | Vrabec 2008 |
| ***CNTNAP3*** | 1 | Sun 2018 | - |
| ***COCH*** | 12 | Fransen 1999, Verhagen 2001, Lemaire 2003, Li 2017, Lopez-Escamez 2018, Gallego-Martinez 2019, Roman-Naranjo 2020 | Usami 2003, Sanchez 2004, Frykholm 2006, Vrabec 2008, Hietikko 2012 |
| ***COL11A1*** | 1 | Roman-Naranjo 2020 | - |
| ***COL11A2*** | 1 | Roman-Naranjo 2020 | - |
| ***COL26A1*** | 1 | Sun 2018 | - |
| ***COL4A6*** | 1 | Roman-Naranjo 2020 | - |
| ***CORIN*** | 1 | Sun 2018 | - |
| ***CPS1*** | 1 | Sun 2018 | - |
| ***CREB3*** | 1 | - | Vrabec 2008 |
| ***CROCC2*** | 1 | Sun 2018 | - |
| ***CRYM*** | 1 | Roman-Naranjo 2020 | - |
| ***CSMD1*** | 1 | Sun 2018 | - |
| ***CSNK1A1L*** | 1 | Sun 2018 | - |
| ***CTLA4*** | 2 | Li 2017 | Lopez-Escamez 2010 |
| ***CTNNA1*** | 1 | - | Gallego-Martinez 2020 |
| ***CTNNA2*** | 1 | - | Gallego-Martinez 2020 |
| ***CTNNB1*** | 1 | - | Gallego-Martinez 2020 |
| ***CTNND1*** | 1 | - | Gallego-Martinez 2020 |
| ***CXCL1*** | 1 | Sun 2018 | - |
| ***CXCL10*** | 2 | Sun 2018, Oh 2020 | - |
| ***CXCL11*** | 1 | Sun 2018 | - |
| ***CXCL12*** | 2 | Sun 2018 | Gallego-Martinez 2020 |
| ***CXCL5*** | 1 | Sun 2018 | - |
| ***CXCL9*** | 2 | Sun 2018 | Oh 2020 |
| ***CXCR2*** | 1 | Sun 2018 | - |
| ***CXCR4*** | 1 | - | Gallego-Martinez 2020 |
| ***CYBA*** | 2 | Teranishi 2013, Gallego-Martinez 2020 | - |
| ***CYP11B2*** | 2 | Sun 2018 | Teggie 2017 |
| ***CYP1A1*** | 1 | - | Huang 2019 |
| ***CYP2B6*** | 1 | Huang 2019 | - |
| ***CYP2F1*** | 1 | Sun 2018 | - |
| ***CYP4A11*** | 1 | Sun 2018 | - |
| ***CYP7A1*** | 1 | - | Teggi 2017 |
| ***CYPF43*** | 1 | Sun 2018 | - |
| ***DCDC2*** | 1 | Roman-Naranjo 2020 | - |
| ***DDIT4*** | 1 | Sun 2018 | - |
| ***DDX3Y*** | 1 | Sun 2018 | - |
| ***DEFB1*** | 1 | Li 2017 | - |
| ***DFNB59*** | 1 | Roman-Naranjo 2020 | - |
| ***DGCR6*** | 1 | Sun 2018 | - |
| ***DGK1*** | 1 | Sun 2018 | - |
| ***DIABLO*** | 1 | Roman-Naranjo 2020 | - |
| ***DIAPH1*** | 3 | Li 2017, Roman-Naranjo 2020 | Frykholm 2006 |
| ***DLC1*** | 1 | Gallego-Martinez 2020 | - |
| ***DMXL2*** | 1 | Roman-Naranjo 2020 | - |
| ***DNM1P33*** | 1 | Sun 2018 | - |
| ***DPT*** | 4 | Martin-Sierra 2017, Lopez-Escamez 2018, Gallego-Martinez 2019, Oh 2020 | - |
| ***DPY5*** | 1 | Sun 2018 | - |
| ***DPYSL2*** | 1 | - | Gallego-Martinez 2020 |
| ***DPYSL5*** | 1 | Gallego-Martinez 2020 | - |
| ***DRC1*** | 1 | Sun 2018 | - |
| ***DRD1*** | 1 | - | Teggi 2017 |
| ***DRD4*** | 1 | Sun 2018 | - |
| ***DTNA*** | 5 | Requena 2015, Li 2017, Lopez-Escamez 2018, Gallego-Martinez 2019, Oh 2020 | - |
| ***EDN2*** | 1 | Sun 2018 | - |
| ***EDNRB*** | 1 | Sun 2018 | - |
| ***EFNA1*** | 1 | - | Gallego-Martinez 2020 |
| ***EFNA5*** | 1 | - | Gallego-Martinez 2020 |
| ***EFNB1*** | 1 | - | Gallego-Martinez 2020 |
| ***EFNB2*** | 1 | - | Gallego-Martinez 2020 |
| ***EFNB3*** | 1 | - | Gallego-Martinez 2020 |
| ***EGR3*** | 1 | Sun 2018 | - |
| ***EIF2AK1*** | 1 | Sun 2018 | - |
| ***ELFN1-AS1*** | 1 | Sun 2018 | - |
| ***ELMOD3*** | 1 | Roman-Naranjo 2020 | - |
| ***ELOVL4*** | 1 | Sun 2018 | - |
| ***ENAH*** | 1 | Gallego-Martinez 2020 | - |
| ***ENPEP*** | 1 | - | Gallego-Martinez 2020 |
| ***ENSG00000130396*** | 1 | - | Gallego-Martinez 2020 |
| ***ENSG00000165197*** | 1 | - | Gallego-Martinez 2020 |
| ***EPB42*** | 1 | Sun 2018 | - |
| ***EPHA1*** | 1 | - | Gallego-Martinez 2020 |
| ***EPHA3*** | 1 | - | Gallego-Martinez 2020 |
| ***EPHA4*** | 1 | - | Gallego-Martinez 2020 |
| ***EPHA5*** | 1 | Gallego-Martinez 2020 | - |
| ***EPHA7*** | 1 | Gallego-Martinez 2020 | - |
| ***EPHB1*** | 1 | Gallego-Martinez 2020 | - |
| ***EPHB2*** | 2 | Sun 2018, Gallego-Martinez 2020 | - |
| ***EPHB4*** | 1 | - | Gallego-Martinez 2020 |
| ***EPO*** | 1 | Sun 2018 | - |
| ***EPS8*** | 1 | Roman-Naranjo 2020 | - |
| ***EPS8L2*** | 1 | Roman-Naranjo 2020 | - |
| ***EREG*** | 1 | Sun 2018 | - |
| ***ESAM*** | 1 | Gallego-Martinez 2020 | - |
| ***ESPN*** | 2 | Gallego-Martinez 2019, Roman-Naranjo 2020 | - |
| ***ESR1*** | 2 | Li 2017 | Nishio 2013 |
| ***ESR2*** | 1 | Li 2017 | - |
| ***ESRP1*** | 1 | Roman-Naranjo 2020 | - |
| ***ESRRB*** | 2 | Gallego-Martinez 2019, Roman-Naranjo 2020 | - |
| ***EYA4*** | 2 | Gallego-Martinez 2019, Roman-Naranjo 2020 | - |
| ***F2*** | 1 | Sun 2018 | - |
| ***F11R*** | 1 | - | Gallego-Martinez 2020 |
| ***F2R*** | 1 | Sun 2018 | - |
| ***F2RL3*** | 1 | Sun 2018 | - |
| ***FAM107B*** | 1 | - | Gallego-Martinez 2019 |
| ***FAM136A*** | 5 | Requena 2015, Li 2017, Lopez-Escamez 2018, Gallego-Martinez 2019, Oh 2020 | - |
| ***FAM3B*** | 1 | Sun 2018 | - |
| ***FAM71E1*** | 1 | Sun 2018 | - |
| ***FAMM166A*** | 1 | Sun 2018 | - |
| ***FCGR36*** | 1 | Sun 2018 | - |
| ***FCHO1*** | 1 | Li 2017 | - |
| ***FCHO2*** | 1 | Li 2017 | - |
| ***FFAR1*** | 1 | Sun 2018 | - |
| ***FGF12*** | 1 | Sun 2018 | - |
| ***FGF2*** | 1 | Sun 2018 | - |
| ***FGFR2*** | 1 | - | Gallego-Martinez 2020 |
| ***FHL1*** | 1 | Sun 2018 | - |
| ***FIBCD1*** | 1 | Sun 2018 | - |
| ***FLNA*** | 1 | Li 2017 | - |
| ***FOLR1*** | 1 | Sun 2018 | - |
| ***FOXE1*** | 1 | Sun 2018 | - |
| ***FPR3*** | 1 | Sun 2018 | - |
| ***FRMPD2B*** | 1 | Sun 2018 | - |
| ***FUT1*** | 1 | Sun 2018 | - |
| ***FYN*** | 1 | - | Gallego-Martinez 2020 |
| ***FZD1*** | 1 | - | Gallego-Martinez 2020 |
| ***FZD10*** | 1 | Gallego-Martinez 2020 | - |
| ***FZD3*** | 1 | - | Gallego-Martinez 2020 |
| ***FZD5*** | 1 | - | Gallego-Martinez 2020 |
| ***FZD6*** | 1 | - | Gallego-Martinez 2020 |
| ***GAB1*** | 2 | Gallego-Martinez 2020, Roman-Naranjo 2020 | - |
| ***GABRB2*** | 1 | Sun 2018 | - |
| ***GALR2*** | 1 | Sun 2018 | - |
| ***GATA2*** | 1 | Sun 2018 | - |
| ***GBP7*** | 1 | Sun 2018 | - |
| ***GCM1*** | 1 | Sun 2018 | - |
| ***GIPC3*** | 1 | Roman-Naranjo 2020 | - |
| ***GJB2*** | 2 | Gallego-Martinez 2019, Roman-Naranjo 2020 | - |
| ***GJB3*** | 2 | Li 2017, Roman-Naranjo 2020 | - |
| ***GJB6*** | 2 | Li 2017, Roman-Naranjo 2020 | - |
| ***GLAST1*** | 1 | Li 2017 | - |
| ***GLDC*** | 1 | Sun 2018 | - |
| ***GLI1*** | 1 | - | Gallego-Martinez 2020 |
| ***GLI3*** | 1 | Gallego-Martinez 2020 | - |
| ***GLIPR1L2*** | 1 | Sun 2018 | - |
| ***GNA14*** | 1 | - | Gallego-Martinez 2020 |
| ***GNAI1*** | 1 | - | Gallego-Martinez 2020 |
| ***GNAI2*** | 1 | - | Gallego-Martinez 2020 |
| ***GNAI3*** | 1 | - | Gallego-Martinez 2020 |
| ***GNAO1*** | 1 | - | Gallego-Martinez 2020 |
| ***GNAS*** | 1 | Gallego-Martinez 2020 | - |
| ***GNB4*** | 1 | - | Gallego-Martinez 2020 |
| ***GNG11*** | 1 | - | Gallego-Martinez 2020 |
| ***GNG2*** | 1 | - | Gallego-Martinez 2020 |
| ***GNG3*** | 1 | Sun 2018 | - |
| ***GNG7*** | 1 | - | Gallego-Martinez 2020 |
| ***GP2*** | 1 | - | Teggi 2017 |
| ***GPER1*** | 1 | Sun 2018 | - |
| ***GPR17*** | 1 | Sun 2018 | - |
| ***GPR20*** | 1 | Sun 2018 | - |
| ***GPR32*** | 1 | Sun 2018 | - |
| ***GPR55*** | 1 | Sun 2018 | - |
| ***GPSM2*** | 1 | Roman-Naranjo 2020 | - |
| ***GPX1*** | 2 | Li 2017 | Teranishi 2012 |
| ***GPX4*** | 1 | - | Oh 2020 |
| ***GPX5*** | 1 | - | Oh 2020 |
| ***GRAP*** | 1 | Roman-Naranjo 2020 | - |
| ***GRHL2*** | 2 | Gallego-Martinez 2019, Roman-Naranjo 2020 | - |
| ***GRIN1*** | 1 | Sun 2018 | - |
| ***GRXCR1*** | 1 | Roman-Naranjo 2020 | - |
| ***GRXCR2*** | 1 | Roman-Naranjo 2020 | - |
| ***GSDME*** | 1 | Roman-Naranjo 2020 | - |
| ***GSK3B*** | 1 | - | Gallego-Martinez 2020 |
| ***GSTA1*** | 1 | Sun 2018 | - |
| ***GSTM1*** | 1 | Sun 2018 | - |
| ***HBA1*** | 1 | Sun 2018 | - |
| ***HBA2*** | 1 | Sun 2018 | - |
| ***HBEGF*** | 1 | Sun 2018 | - |
| ***HCAR2*** | 1 | Sun 2018 | - |
| ***HCAR3*** | 1 | Sun 2018 | - |
| ***HCFC1*** | 4 | Vrabec 2008, Hietikko 2012, Li 2017 | Oh 2020 |
| ***HCG9*** | 1 | Sun 2018 | - |
| ***HCRT*** | 1 | Sun 2018 | - |
| ***HES4*** | 1 | Sun 2018 | - |
| ***HFE2*** | 1 | Sun 2018 | - |
| ***HFM1*** | 1 | Sun 2018 | - |
| ***HGF*** | 1 | Roman-Naranjo 2020 | - |
| ***HIF3A*** | 1 | Sun 2018 | - |
| ***HLA-A*** | 6 | Morrison 1994, Li 2017, Chan 2018 | Koyama 1993, Melchiorri 2002, Yeo 2002 |
| ***HLA-B*** | 8 | Yeo 2002, Rawal 2010, Li 2017 | Koyama 1993, Morrison 1994, Melchorri 2002, Gazquez 2012, Chan 2018 |
| ***HLA-C*** | 8 | Morrison 1994, Melchorri 2002, Yeo 2002, Khorsandi 2011, Li 2017 | Koyama 1993, Chan 2018, Oh 2020 |
| ***HLA-DPA2*** | 1 |  | - |
| ***HLA-DQA1*** | 4 | Li 2017 | Koyama 1993, Yeo 2002, Chan 2018 |
| ***HLA-DQB1*** | 4 | - | Koyama 1993, Yeo 2002, Lopez-Escamez 2007, Chan 2018 |
| ***HLA-DQB2*** | 3 | Sun 2018 | Koyama 1993, Chan 2018 |
| ***HLA-DRB1*** | 8 | Koyama 1993, Yeo 2002, Koo 2003, Lopez-Escamez 2002, Lopez-Escamez 2007, Li 2017 | Melchiorri 2002, Oh 2020 |
| ***HMOX1*** | 1 | Sun 2018 | - |
| ***HMX2*** | 2 | Skarp 2019 | - |
| ***HNF17*** | 1 | Sun 2018 | - |
| ***HOMER2*** | 1 | Roman-Naranjo 2020 | - |
| ***HRH4*** | 2 | Sun 2018, Qin 2019 | - |
| ***HSD3B1*** | 1 | - | Teggi 2017 |
| ***HSPA1A*** | 3 | Kawaguchi 2008, Li 2017 | Oh 2020 |
| ***HTR2A*** | 2 | Sun 2018 | Vrabec 2008 |
| ***ICAM1*** | 2 | Gallego-Martinez 2020 | Vrabec 2008 |
| ***IFI6*** | 1 | Sun 2018 | - |
| ***IFITM4P*** | 1 | Sun 2018 | - |
| ***IFNG*** | 3 | Li 2017 | Vrabec 2008, Gazquez 2013 |
| ***IFNLR1*** | 1 | Roman-Naranjo 2020 | - |
| ***IGF1*** | 1 | - | Gallego-Martinez 2020 |
| ***IL1A*** | 3 | Furuta 2011, Li 2017 | Oh 2020 |
| ***IL1B*** | 3 | Li 2017, Lopez-Escamez 2018 | Furuta 2011 |
| ***IL1R1*** | 1 | - | Oh 2020 |
| ***IL6*** | 4 | Li 2017, Lopez-Escamez 2018 | Vrabec 2008, Oh 2020 |
| ***IL9*** | 1 | - | Vrabec 2008 |
| ***IL10*** | 2 | - | Vrabec 2008, Oh 2020 |
| ***IL17D*** | 1 | Sun 2018 | - |
| ***IL18*** | 1 | - | Oh 2020 |
| ***IL23R*** | 1 | Sun 2018 | - |
| ***ILK*** | 1 | - | Gallego-Martinez 2020 |
| ***IRS1*** | 1 | Gallego-Martinez 2020 | - |
| ***IRS2*** | 1 | - | Gallego-Martinez 2020 |
| ***ITGA3*** | 1 | - | Gallego-Martinez 2020 |
| ***ITGA4*** | 1 | - | Gallego-Martinez 2020 |
| ***ITGB1*** | 1 | - | Gallego-Martinez 2020 |
| ***ITK*** | 1 | - | Gallego-Martinez 2020 |
| ***JAK1*** | 1 | - | Vrabec 2008 |
| ***JAM2*** | 1 | Gallego-Martinez 2020 | - |
| ***JAM3*** | 1 | - | Gallego-Martinez 2020 |
| ***JPH4*** | 1 | Sun 2018 | - |
| ***KARS*** | 1 | Roman-Naranjo 2020 | - |
| ***KCNA1*** | 1 | Li 2017 | - |
| ***KCNA5*** | 1 | Sun 2018 | - |
| ***KCNE1*** | 8 | Doi 2005, Hietikko 2012, Lopes 2016, Dai 2019 | Vrabec 2008, Campbell 2010, Gallego-Martinez 2019, Oh 2020 |
| ***KCNE3*** | 7 | Doi 2005, Li 2017, Dai 2019 | Campbell 2010, Hietikko 2012, Gallego-Martinez 2019, Oh 2020 |
| ***KCNJ2*** | 1 | Sun 2018 | - |
| ***KCNJ10*** | 1 | Gallego-Martinez 2019 | - |
| ***KCNQ1*** | 1 | Gallego-Martinez 2019 | - |
| ***KCNQ4*** | 3 | Li 2017, Roman-Naranjo 2020 | Gallego-Martinez 2019 |
| ***KDM6D*** | 1 | Sun 2018 | - |
| ***KEL*** | 1 | Sun 2018 | - |
| ***KIF7*** | 1 | Sun 2018 | - |
| ***KITLG*** | 1 | Roman-Naranjo 2020 | - |
| ***KMT2C*** | 1 | - | Mehrjoo 2020 |
| ***KRAS*** | 1 | - | Gallego-Martinez 2020 |
| ***KRT17P1*** | 1 | Sun 2018 | - |
| ***KRT23*** | 11 | Sun 2018 | - |
| ***KRTSP42*** | 1 | Sun 2018 | - |
| ***KY*** | 1 | Sun 2018 | - |
| ***LCN2*** | 1 | Sun 2018 | - |
| ***LEP*** | 1 | Sun 2018 | - |
| ***LETM2*** | 1 | Sun 2018 | - |
| ***LHFPL5*** | 1 | Roman-Naranjo 2020 | - |
| ***LIMK1*** | 1 | - | Gallego-Martinez 2020 |
| ***LIMK2*** | 1 | Gallego-Martinez 2020 | - |
| ***LINGO1*** | 1 | - | Gallego-Martinez 2020 |
| ***LIPC*** | 1 | Sun 2018 | - |
| ***LMX1A*** | 1 | Roman-Naranjo 2020 | - |
| ***LOXHD1*** | 1 | Roman-Naranjo 2020 | - |
| ***LPAR4*** | 1 | Sun 2018 | - |
| ***LRRC4*** | 1 | Gallego-Martinez 2020 | - |
| ***LRRC4C*** | 1 | - | Gallego-Martinez 2020 |
| ***LRTOMT*** | 1 | Roman-Naranjo 2020 | - |
| ***LSAMP*** | 1 | Mehrjoo 2020 | - |
| ***LSS*** | 1 | - | Teggi 2017 |
| ***LTF*** | 1 | Li 2017 | - |
| ***LUM*** | 1 | Sun 2018 | - |
| ***LYPD2*** | 1 | Sun 2018 | - |
| ***MAP7D2*** | 1 | Sun 2018 | - |
| ***MAPK1*** | 1 | - | Gallego-Martinez 2020 |
| ***MAPK10*** | 1 | - | Gallego-Martinez 2020 |
| ***MAPK11*** | 1 | - | Gallego-Martinez 2020 |
| ***MAPK12*** | 1 | - | Gallego-Martinez 2020 |
| ***MAPK13*** | 1 | - | Gallego-Martinez 2020 |
| ***MAPK14*** | 1 | - | Gallego-Martinez 2020 |
| ***MARCO*** | 1 | Sun 2018 | - |
| ***MARVELD2*** | 2 | Gallego-Martinez 2019, Roman-Naranjo 2020 | - |
| ***MCHR1*** | 1 | Sun 2018 | - |
| ***MCM2*** | 1 | Roman-Naranjo 2020 | - |
| ***MCUR1*** | 1 | Sun 2018 | - |
| ***MET*** | 1 | Roman-Naranjo 2020 | - |
| ***METTL7B*** | 1 | Sun 2018 | - |
| ***MICA*** | 3 | Gazquez 2012, Li 2017 | Gallego-Martinez 2019, Oh 2020 |
| ***MICD*** | 1 | Sun 2018 | - |
| ***MIF*** | 4 | Yazdani 2013 | Gazquez 2013, Gallego-Martinez 2019, Oh 2020 |
| ***MIRN96*** | 1 | Roman-Naranjo 2020 | - |
| ***MLLT4*** | 1 | Gallego-Martinez 2020 | - |
| ***MME*** | 2 | Sun 2018 | Gallego-Martinez 2020 |
| ***MMP1*** | 1 | Sun 2018 |  |
| ***MMP11*** | 1 | - | Gallego-Martinez 2020 |
| ***MMP14*** | 1 | - | Gallego-Martinez 2020 |
| ***MMP15*** | 1 | - | Gallego-Martinez 2020 |
| ***MMP16*** | 1 | - | Gallego-Martinez 2020 |
| ***MMP2*** | 1 | - | Gallego-Martinez 2020 |
| ***MMP24*** | 1 | - | Gallego-Martinez 2020 |
| ***MMP9*** | 1 | - | Gallego-Martinez 2020 |
| ***MPZL2*** | 1 | Roman-Naranjo 2020 | - |
| ***MRAS*** | 1 | - | Gallego-Martinez 2020 |
| ***MRPS12*** | 2 | Li 2017 | Pacheu-Grau 2012 |
| ***MRPS36P4*** | 1 | Sun 2018 | - |
| ***MSR1*** | 1 | Sun 2018 | - |
| ***MSRB3*** | 2 | Gallego-Martinez 2019, Roman-Naranjo 2020 | - |
| ***MSN*** | 1 | - | Gallego-Martinez 2020 |
| ***MT1X*** | 1 | Sun 2018 | - |
| ***MT2A*** | 1 | Sun 2018 | - |
| ***MT2P1*** | 1 | Sun 2018 | - |
| ***MT3*** | 1 | Sun 2018 | - |
| ***MT5178*** | 1 | - | Teranishi 2013 |
| ***MTHFR*** | 3 | Huang 2013, Li 2017, Oh 2020 | - |
| ***MTNR1B*** | 2 | Li 2017 | Teranishi 2013 |
| ***MTR*** | 2 | Li 2017 | Teranishi 2013 |
| ***MTRR*** | 2 | Li 2017 | Teranishi 2013 |
| ***MT-RNR1*** | 1 | - | Pacheu-Grau 2012 |
| ***MT-RNR2*** | 1 | - | Pacheu-Grau 2012 |
| ***MYBPH*** | 1 | Sun 2018 | - |
| ***MYD88*** | 1 | - | Oh 2020 |
| ***MYH14*** | 2 | Gallego-Martinez 2019, Roman-Naranjo 2020 | - |
| ***MYH9*** | 1 | Roman-Naranjo 2020 | - |
| ***MYL2*** | 1 | - | Gallego-Martinez 2020 |
| ***MYL4*** | 1 | Sun 2018 | - |
| ***MYL9*** | 1 | - | Gallego-Martinez 2020 |
| ***MYLPF*** | 1 | - | Gallego-Martinez 2020 |
| ***MYO15A*** | 1 | Roman-Naranjo 2020 | - |
| ***MYO3A*** | 1 | Roman-Naranjo 2020 | - |
| ***MYO6*** | 1 | Roman-Naranjo 2020 | - |
| ***MYO7A*** | 2 | Gallego-Martinez 2019, Roman-Naranjo 2020 | - |
| ***MYOC*** | 1 | Sun 2018 | - |
| ***MYRFL*** | 1 | Sun 2018 | - |
| ***NAGA*** | 1 | Li 2017 | - |
| ***NARS2*** | 1 | Roman-Naranjo 2020 | - |
| ***NBF13P*** | 1 | Sun 2018 | - |
| ***NCMAP*** | 1 | Sun 2018 | - |
| ***NCK1*** | 1 | Gallego-Martinez 2020 | - |
| ***NDUFS2*** | 1 | Li 2017 | - |
| ***NECAB2*** | 1 | Sun 2018 | - |
| ***NEDD4L*** | 1 | - | Teggi 2017 |
| ***NFATC2*** | 1 | - | Gallego-Martinez 2020 |
| ***NFATC3*** | 1 | Gallego-Martinez 2020 | - |
| ***NFATC4*** | 1 | - | Gallego-Martinez 2020 |
| ***NFKB1*** | 7 | Cabrera 2014, Frejo 2017, Li 2017, Lopez-Escamez 2018, Gallego-Martinez 2019, Oh 2020 | Vrabec 2008 |
| ***NGFB*** | 1 | - | Vrabec 2008 |
| ***NGFR*** | 1 | - | Gallego-Martinez 2020 |
| ***NKAIN2*** | 2 | Sun 2018 | Teggi 2017 |
| ***NKAIN3*** | 1 | - | Teggi 2017 |
| ***NLRP12*** | 1 | Requena 2019 | - |
| ***NLRP3*** | 1 | Roman-Naranjo 2020 | - |
| ***NOL3*** | 1 | Sun 2018 | - |
| ***NOS1*** | 1 | - | Gazquez 2011 |
| ***NOS2*** | 2 | Li 2017 | Gazquez 2011 |
| ***NOS3*** | 3 | Li 2017, Oh 2020 | Teranishi 2013 |
| ***NOTCH2*** | 1 | Oh 2020 | - |
| ***NOTCH3*** | 1 | Li 2017 | - |
| ***NOTUM*** | 1 | - | Gallego-Martinez 2020 |
| ***NOX3*** | 1 | Gallego-Martinez 2020 | - |
| ***NPAP1P2*** | 1 | Sun 2018 | - |
| ***NPM1P35*** | 1 | Sun 2018 | - |
| ***NPNT*** | 1 | Sun 2018 | - |
| ***NPR1*** | 2 | Li 2017, Sun 2018 | - |
| ***NPR2*** | 1 | Li 2017 | - |
| ***NPR3*** | 1 | Li 2017 | - |
| ***NR2C2*** | 1 | - | Vrabec 2008 |
| ***NR3B2*** | 1 | - | Gallego-Martinez 2019 |
| ***NRARP*** | 1 | Sun 2018 | - |
| ***NRAS*** | 1 | - | Gallego-Martinez 2020 |
| ***NRG1*** | 1 | Sun 2018 | - |
| ***NRP1*** | 1 | - | Gallego-Martinez 2020 |
| ***NSFP1*** | 1 | Sun 2018 | - |
| ***NTN3*** | 1 | - | Gallego-Martinez 2020 |
| ***NTN4*** | 1 | Gallego-Martinez 2020 | - |
| ***NTNG1*** | 1 | - | Gallego-Martinez 2020 |
| ***NTNG2*** | 1 | - | Gallego-Martinez 2020 |
| ***NTRK2*** | 1 | - | Gallego-Martinez 2020 |
| ***NTRK3*** | 1 | - | Gallego-Martinez 2020 |
| ***NTSR1*** | 1 | Sun 2018 | - |
| ***OCLN*** | 1 | - | Gallego-Martinez 2020 |
| ***OLFM1*** | 1 | Sun 2018 | - |
| ***OR3A3*** | 1 | Sun 2018 | - |
| ***OR5H2*** | 1 | - | Mehrjoo 2020 |
| ***OR5K4*** | 1 | - | Mehrjoo 2020 |
| ***OSBPL2*** | 1 | Roman-Naranjo 2020 | - |
| ***OSTCP5*** | 1 | Roman-Naranjo 2020 | - |
| ***OTOA*** | 1 | Roman-Naranjo 2020 | - |
| ***OTOF*** | 1 | Roman-Naranjo 2020 | - |
| ***OTOG*** | 1 | Roman-Naranjo 2020 | - |
| ***OTOGL*** | 1 | Roman-Naranjo 2020 | - |
| ***OXTR*** | 1 | Sun 2018 | - |
| ***P2RX2*** | 2 | Gallego-Martinez 2019, Roman-Naranjo 2020 | - |
| ***P2RX7*** | 1 | Sun 2018 | - |
| ***P2RY2*** | 1 | Sun 2018 | - |
| ***PACERR*** | 1 | Sun 2018 | - |
| ***PAK1*** | 1 | - | Gallego-Martinez 2020 |
| ***PAK2*** | 1 | - | Gallego-Martinez 2020 |
| ***PAK3*** | 1 | - | Gallego-Martinez 2020 |
| ***PAK4*** | 1 | - | Gallego-Martinez 2020 |
| ***PAK6*** | 1 | - | Gallego-Martinez 2020 |
| ***PALM*** | 1 | Sun 2018 | - |
| ***PAPPA2*** | 1 | - | Gallego-Martinez 2020 |
| ***PARD3*** | 1 | - | Gallego-Martinez 2020 |
| ***PARD6A*** | 1 | - | Gallego-Martinez 2020 |
| ***PARD6G*** | 1 | - | Gallego-Martinez 2020 |
| ***PARP1*** | 3 | Lopez-Escamez 2009, Li 2017 | Oh 2020 |
| ***PCDH15*** | 1 | Roman-Naranjo 2020 | - |
| ***PCDHGA12*** | 1 | Sun 2018 | - |
| ***PDE1C*** | 1 | Roman-Naranjo 2020 | - |
| ***PDGFA*** | 1 | - | Gallego-Martinez 2020 |
| ***PDGFC*** | 1 | - | Gallego-Martinez 2020 |
| ***PDK1*** | 1 | - | Gallego-Martinez 2020 |
| ***PDK4*** | 1 | Sun 2018 | - |
| ***PDZD7*** | 1 | Roman-Naranjo 2020 | - |
| ***PECAM1*** | 1 | - | Gallego-Martinez 2020 |
| ***PER1*** | 1 | Sun 2018 | - |
| ***PGBD4P3*** | 1 | Sun 2018 | - |
| ***PIK3C2G*** | 2 | Klar 2006, Gabrikova 2010 | - |
| ***PIK3CA*** | 1 | Gallego-Martinez 2020 | - |
| ***PIK3CB*** | 1 | - | Gallego-Martinez 2020 |
| ***PIK3R1*** | 1 | - | Gallego-Martinez 2020 |
| ***PIK3R3*** | 1 | - | Gallego-Martinez 2020 |
| ***PJVK*** | 1 | Roman-Naranjo 2020 | - |
| ***PKD2*** | 1 | - | Teggi 2017 |
| ***PLCE1*** | 1 | Gallego-Martinez 2020 | - |
| ***PLCH1*** | 1 | - | Gallego-Martinez 2020 |
| ***PLCH2*** | 1 | - | Gallego-Martinez 2020 |
| ***PLEK2*** | 1 | Sun 2018 | - |
| ***PLXNA2*** | 1 | - | Gallego-Martinez 2020 |
| ***PLXNA3*** | 1 | Gallego-Martinez 2020 | - |
| ***PLXNA4*** | 1 | Gallego-Martinez 2020 | - |
| ***PLXNB1*** | 1 | Gallego-Martinez 2020 | - |
| ***PLXNB2*** | 1 | Gallego-Martinez 2020 | - |
| ***PLXNC1*** | 1 | Gallego-Martinez 2020 | - |
| ***PLXND1*** | 1 | Gallego-Martinez 2020 | - |
| ***PNPT1*** | 2 | Roman-Naranjo 2020 | Gallego-Martinez 2019 |
| ***POLB*** | 1 | - | Vrabec 2008 |
| ***PON1*** | 2 | Li 2017 | Teranishi 2012 |
| ***PON2*** | 2 | Li 2017 | Teranishi 2012 |
| ***POU2F1*** | 1 | - | Vrabec 2008 |
| ***POU3F4*** | 1 | Roman-Naranjo 2020 | - |
| ***POU4F3*** | 5 | Li 2017, Gallego-Martinez 2019, Roman-Naranjo 2020 | Frykholm 2006, Vrabec 2008 |
| ***PPIP5K2*** | 1 | Roman-Naranjo 2020 | - |
| ***PPP1R17*** | 1 | Sun 2018 | - |
| ***PPP3CA*** | 1 | - | Gallego-Martinez 2020 |
| ***PPP3CB*** | 1 | - | Gallego-Martinez 2020 |
| ***PPP3CC*** | 1 | - | Gallego-Martinez 2020 |
| ***PPP3R1*** | 1 | - | Gallego-Martinez 2020 |
| ***PRDX2P4*** | 1 | Sun 2018 | - |
| ***PRKACB*** | 1 | - | Gallego-Martinez 2020 |
| ***PRKAR2A*** | 1 | - | Gallego-Martinez 2020 |
| ***PRKAR2B*** | 1 | - | Gallego-Martinez 2020 |
| ***PRKCA*** | 1 | - | Gallego-Martinez 2020 |
| ***PRKCB*** | 5 | Martin-Sierra 2016, Lopez-Escamez 2018, Gallego-Martinez 2019 | Gallego-Martinez 2020, Oh 2020 |
| ***PRKCZ*** | 1 | - | Gallego-Martinez 2020 |
| ***PRKD1*** | 1 | Sun 2018 | - |
| ***PRKD3*** | 1 | Gallego-Martinez 2020 | - |
| ***PRKG1*** | 1 | - | Teggi 2017 |
| ***PRKY*** | 1 | Sun 2018 | - |
| ***PRPS1*** | 1 | Roman-Naranjo 2020 | - |
| ***PRSS35*** | 1 | Sun 2018 | - |
| ***PSPHP1*** | 1 | Sun 2018 | - |
| ***PTCH1*** | 1 | - | Gallego-Martinez 2020 |
| ***PTGS2*** | 1 | - | Oh 2020 |
| ***PTH1R*** | 1 | Sun 2018 | - |
| ***PTK2*** | 1 | Gallego-Martinez 2020 | - |
| ***PTO52*** | 1 | Sun 2018 | - |
| ***PTPN11*** | 1 | - | Gallego-Martinez 2020 |
| ***PTPN22*** | 3 | Lopez-Escamez 2010, Li 2017, Oh 2020 | - |
| ***PTPRQ*** | 1 | Roman-Naranjo 2020 | - |
| ***PVALB*** | 1 | Sun 2018 | - |
| ***PVRL2*** | 1 | - | Vrabec 2008 |
| ***PXN*** | 1 | - | Gallego-Martinez 2020 |
| ***RAB39A*** | 1 | Sun 2018 | - |
| ***RAC1*** | 1 | - | Gallego-Martinez 2020 |
| ***RAF1*** | 1 | Gallego-Martinez 2020 | - |
| ***RAMP3*** | 1 | Sun 2018 | - |
| ***RAP1A*** | 1 | - | Gallego-Martinez 2020 |
| ***RAP1B*** | 1 | - | Gallego-Martinez 2020 |
| ***RAP1GAP*** | 1 | - | Gallego-Martinez 2020 |
| ***RAPGEF3*** | 1 | - | Gallego-Martinez 2020 |
| ***RAPGEF4*** | 1 | Gallego-Martinez 2020 | - |
| ***RASA1*** | 1 | - | Gallego-Martinez 2020 |
| ***RDX*** | 2 | Gallego-Martinez 2019, Roman-Naranjo 2020 | - |
| ***REL*** | 2 | Li 2017 | Cabrera 2014 |
| ***RENBP*** | 1 | Vrabec 2008 | - |
| ***RERGL*** | 1 | - | Gabrikova 2010 |
| ***REST*** | 1 | Roman-Naranjo 2020 | - |
| ***RGN*** | 1 | Sun 2018 | - |
| ***RGS3*** | 1 | Gallego-Martinez 2020 | - |
| ***RHOA*** | 1 | - | Gallego-Martinez 2020 |
| ***RHOD*** | 1 | - | Gallego-Martinez 2020 |
| ***RIPOR2*** | 1 | Roman-Naranjo 2020 | - |
| ***ROBO1*** | 1 | - | Gallego-Martinez 2020 |
| ***ROBO2*** | 1 | Gallego-Martinez 2020 | - |
| ***ROCK1*** | 1 | - | Gallego-Martinez 2020 |
| ***ROCK2*** | 1 | - | Gallego-Martinez 2020 |
| ***ROR1*** | 1 | Roman-Naranjo 2020 | - |
| ***ROR2*** | 1 | Sun 2018 | - |
| ***RPS4Y1*** | 1 | Sun 2018 | - |
| ***RPSAP47*** | 1 | Sun 2018 | - |
| ***RRAS*** | 1 | - | Gallego-Martinez 2020 |
| ***RSPH1*** | 1 | Sun 2018 | - |
| ***RYK*** | 1 | - | Gallego-Martinez 2020 |
| ***RYR2*** | 1 | Sun 2018 | - |
| ***S100A14*** | 1 | Sun 2018 | - |
| ***S1PR2*** | 1 | Roman-Naranjo 2020 | - |
| ***SCARA5*** | 1 | Sun 2018 | - |
| ***SCN7A*** | 1 | Sun 2018 | - |
| ***SCO2*** | 1 | Sun 2018 | - |
| ***SCRT2*** | 1 | Sun 2018 | - |
| ***SDC2*** | 1 | - | Gallego-Martinez 2020 |
| ***SDCBP*** | 1 | - | Gallego-Martinez 2020 |
| ***SEMA3A*** | 1 | Gallego-Martinez 2020 | - |
| ***SEMA3B*** | 1 | - | Gallego-Martinez 2020 |
| ***SEMA3C*** | 1 | Gallego-Martinez 2020 | - |
| ***SEMA3D*** | 4 | Martin-Sierra 2017, Lopez-Escamez 2018, Gallego-Martinez 2019 | Oh 2020 |
| ***SEMA3E*** | 1 | - | Gallego-Martinez 2020 |
| ***SEMA3F*** | 1 | - | Gallego-Martinez 2020 |
| ***SEMA4B*** | 1 | Gallego-Martinez 2020 | - |
| ***SEMA4C*** | 1 | - | Gallego-Martinez 2020 |
| ***SEMA4D*** | 1 | - | Gallego-Martinez 2020 |
| ***SEMA4F*** | 1 | Gallego-Martinez 2020 | - |
| ***SEMA4G*** | 1 | - | Gallego-Martinez 2020 |
| ***SEMA5A*** | 1 | - | Gallego-Martinez 2020 |
| ***SEMA5B*** | 1 | - | Gallego-Martinez 2020 |
| ***SEMA6A*** | 1 | - | Gallego-Martinez 2020 |
| ***SEMA6B*** | 2 | Sun 2018 | Gallego-Martinez 2020 |
| ***SEMA6D*** | 1 | Gallego-Martinez 2020 | - |
| ***SEMA7A*** | 1 | - | Gallego-Martinez 2020 |
| ***SERPINB6*** | 1 | Roman-Naranjo 2020 | - |
| ***SETD9*** | 1 | - | Vrabec 2008 |
| ***SGK1*** | 1 | Sun 2018 | - |
| ***SHC1*** | 1 | Gallego-Martinez 2020 | - |
| ***SHROOM3*** | 1 | Sun 2018 | - |
| ***SIGLEC*** | 1 | Sun 2018 | - |
| ***SIGLEC1*** | 1 | Sun 2018 | - |
| ***SIGLEC29P*** | 1 | Sun 2018 | - |
| ***SIK1*** | 3 | Li 2017, Teggi 2017 | Oh 2020 |
| ***SIX1*** | 1 | Roman-Naranjo 2020 | - |
| ***SLC11A1*** | 1 | Sun 2018 | - |
| ***SLC12A1*** | 3 | - | Vrabec 2008, Teggi 2017, Asmar 2018 |
| ***SLC12A2*** | 2 | Li 2017, Gallego-Martinez 2019 | - |
| ***SLC12A3*** | 1 | - | Teggi 2017 |
| ***SLC12A5*** | 1 | Sun 2018 | - |
| ***SLC17A8*** | 1 | Roman-Naranjo 2020 | - |
| ***SLC22A4*** | 1 | Roman-Naranjo 2020 | - |
| ***SLC24A3*** | 2 | Sun 2018 | Teggi 2017 |
| ***SLC25A24P2*** | 1 | Sun 2018 | - |
| ***SLC25A27*** | 1 | Sun 2018 | - |
| ***SLC26A4*** | 5 | Yoshida 2015, Li 2017, Gallego-Martinez 2019, Roman-Naranjo 2020, Tsukada 2020 | - |
| ***SLC26A5*** | 2 | Sun 2018, Roman-Naranjo 2020 | - |
| ***SLC26A7*** | 1 | Sun 2018 | - |
| ***SLC2A3*** | 1 | Sun 2018 | - |
| ***SLC34A2*** | 1 | Sun 2018 | - |
| ***SLC35G1*** | 1 | Sun 2018 | - |
| ***SLC44A2*** | 2 | Nair 2016, Oh 2020 | - |
| ***SLC4A1*** | 1 | Sun 2018 | - |
| ***SLC4A10*** | 1 | Sun 2018 | - |
| ***SLC4A3*** | 1 | Sun 2018 | - |
| ***SLC7A8*** | 1 | Sun 2018 | - |
| ***SLC8A1*** | 3 | Li 2017, Teggi 2017 | Oh 2020 |
| ***SLC8A2*** | 1 | Sun 2018 | - |
| ***SLC8A3*** | 1 | Sun 2018 | - |
| ***SLC9A2*** | 1 | Sun 2018 | - |
| ***SLC9C1*** | 1 | Sun 2018 | - |
| ***SLC9C2*** | 1 | Sun 2018 | - |
| ***SLIT2*** | 1 | Gallego-Martinez 2020 | - |
| ***SLIT3*** | 1 | - | Gallego-Martinez 2020 |
| ***SMIM2*** | 1 | Sun 2018 | - |
| ***SMO*** | 1 | - | Gallego-Martinez 2020 |
| ***SMPX*** | 1 | Roman-Naranjo 2020 | - |
| ***SNCA*** | 1 | Sun 2018 | - |
| ***SNORA70*** | 1 | Sun 2018 | - |
| ***SNORD3B-1*** | 1 | Sun 2018 | - |
| ***SOD2*** | 2 | Li 2017 | Teranishi 2012 |
| ***SORC53*** | 1 | Sun 2018 | - |
| ***SPNS2*** | 1 | Roman-Naranjo 2020 | - |
| ***SPX*** | 1 | Sun 2018 | - |
| ***SRGAP1*** | 1 | - | Gallego-Martinez 2020 |
| ***SRGAP2*** | 1 | Gallego-Martinez 2020 | - |
| ***SRGAP3*** | 1 | - | Gallego-Martinez 2020 |
| ***SSH1*** | 1 | - | Gallego-Martinez 2020 |
| ***SSH3*** | 1 | Gallego-Martinez 2020 | - |
| ***STAT1*** | 1 | - | Vrabec 2008 |
| ***STAT3*** | 1 | - | Vrabec 2008 |
| ***STAT5*** | 1 | - | Vrabec 2008 |
| ***STEAP2*** | 1 | Sun 2018 | - |
| ***STEAP3*** | 1 | Sun 2018 | - |
| ***STEAP4*** | 1 | Sun 2018 | - |
| ***STK36*** | 1 | Gallego-Martinez 2020 | - |
| ***STRC*** | 1 | Roman-Naranjo 2020 | - |
| ***SYNE4*** | 1 | Roman-Naranjo 2020 | - |
| ***SYTL5*** | 1 | Sun 2018 | - |
| ***TBC1D24*** | 1 | Roman-Naranjo 2020 | - |
| ***TBC1D3F*** | 1 | Sun 2018 | - |
| ***TCEB3CL2*** | 1 | Sun 2018 | - |
| ***TCN2*** | 1 | Sun 2018 | - |
| ***TCP10L*** | 1 | Sun 2018 | - |
| ***TECTA*** | 1 | Roman-Naranjo 2020 | - |
| ***TFB1M*** | 2 | Li 2017 | Pacheu-Grau 2012 |
| ***TGFB2*** | 1 | Sun 2018 | - |
| ***TGM2*** | 1 | Sun 2018 | - |
| ***THAP1*** | 1 | - | Gallego-Martinez 2019 |
| ***THCAT158*** | 1 | Sun 2018 | - |
| ***THY1*** | 1 | - | Gallego-Martinez 2020 |
| ***TIMP2*** | 1 | - | Gallego-Martinez 2020 |
| ***TIMP3*** | 1 | - | Gallego-Martinez 2020 |
| ***TJP2*** | 2 | Gallego-Martinez 2019, Roman-Naranjo 2020 | - |
| ***TLR10*** | 4 | Requena 2013, Li 2017 | Gallego-Martinez 2019, Oh 2020 |
| ***TLR2*** | 1 | Oh 2020 | - |
| ***TLR3*** | 2 | Li 2017 | Requena 2013 |
| ***TLR4*** | 1 | Li 2017 | - |
| ***TLR7*** | 2 | Li 2017 | Requena 2013 |
| ***TLR8*** | 2 | Li 2017 | Requena 2013 |
| ***TLR9*** | 1 | - | Oh 2020 |
| ***TM4SF1*** | 1 | Sun 2018 | - |
| ***TMC1*** | 1 | Roman-Naranjo 2020 | - |
| ***TMEM132E*** | 1 | Roman-Naranjo 2020 | - |
| ***TMEM176A*** | 1 | Sun 2018 | - |
| ***TMEM176B*** | 1 | Sun 2018 | - |
| ***TMEM178A*** | 1 | Sun 2018 | - |
| ***TMEM51*** | 1 | Sun 2018 | - |
| ***TMEM55B*** | 1 | Skarp 2019 | - |
| ***TMEM64*** | 1 | Sun 2018 | - |
| ***TMIE*** | 1 | Roman-Naranjo 2020 | - |
| ***TMPRSS3*** | 1 | Roman-Naranjo 2020 | - |
| ***TMPRSS4*** | 1 | Roman-Naranjo 2020 | - |
| ***TMSD4Y*** | 1 | Sun 2018 | - |
| ***TNC*** | 1 | Roman-Naranjo 2020 | - |
| ***TNF*** | 6 | Frejo 2017, Li 2017, Lopez-Escamez 2018 | Vrabec 2008, Gazquez 2013, Oh 2020 |
| ***TNFAIP3*** | 2 | Li 2017 | Cabrera 2014 |
| ***TNFRSF12A*** | 1 | Frejo 2017 | - |
| ***TNFSF11*** | 1 | Sun 2018 | - |
| ***TNFSF12*** | 1 | Frejo 2017 | - |
| ***TNFRSF1A*** | 1 | - | Vrabec 2008 |
| ***TNFRSF1B*** | 1 | - | Vrabec 2008 |
| ***TNIP1*** | 2 | Li 2017 | Cabrera 2014 |
| ***TNNI3*** | 2 | Cabrera 2014, Sun 2018 | - |
| ***TNNT2*** | 1 | Sun 2018 | - |
| ***TPO*** | 1 | Sun 2018 | - |
| ***TPRN*** | 2 | Gallego-Martinez 2019, Roman-Naranjo 2020 | - |
| ***TPSAB1*** | 1 | Sun 2018 | - |
| ***TPTE2P6*** | 1 | Sun 2018 | - |
| ***TRBV6*** | 1 | Sun 2018 | - |
| ***TRIOBP*** | 2 | Roman-Naranjo 2020 | Gallego-Martinez 2019 |
| ***TRPC1*** | 1 | - | Gallego-Martinez 2020 |
| ***TRPC3*** | 1 | - | Gallego-Martinez 2020 |
| ***TRPC6*** | 3 | Sun 2018 | Teggi 2017, Gallego-Martinez 2020 |
| ***TRPM8*** | 1 | Sun 2018 | - |
| ***TRPV1*** | 1 | - | Vrabec 2008 |
| ***TRPV4*** | 2 | Kumagami 2009, Asmar 2018 | - |
| ***TRRAP*** | 1 | Roman-Naranjo 2020 | - |
| ***TSPEAR*** | 1 | Roman-Naranjo 2020 | - |
| ***TTTY14*** | 1 | Sun 2018 | - |
| ***TUBA4A*** | 1 | - | Gallego-Martinez 2020 |
| ***TUBB3*** | 1 | Sun 2018 | - |
| ***TUBB2B*** | 1 | - | Gallego-Martinez 2020 |
| ***TUBB4A*** | 1 | - | Gallego-Martinez 2020 |
| ***TUBB4B*** | 1 | - | Gallego-Martinez 2020 |
| ***TUBB6*** |  |  |  |
| ***TXLNGY*** | 1 | Sun 2018 | - |
| ***UBE2L3*** | 1 | - | Cabrera 2014 |
| ***UNC5B*** | 1 | Sun 2018 | - |
| ***UNC5C*** | 1 | Gallego-Martinez 2020 | - |
| ***UNC5D*** | 1 | - | Gallego-Martinez 2020 |
| ***USH1C*** | 2 | Gallego-Martinez 2019, Roman-Naranjo 2020 | - |
| ***USH1G*** | 1 | Gallego-Martinez 2019 | - |
| ***USP3*** | 1 | Mehrjoo 2020 | - |
| ***UTF1*** | 1 | Sun 2018 | - |
| ***UTY*** | 1 | Sun 2018 | - |
| ***VASP*** | 1 | - | Gallego-Martinez 2020 |
| ***VAV1*** | 1 | - | Gallego-Martinez 2020 |
| ***VAV2*** | 1 | Gallego-Martinez 2020 | - |
| ***VAV3*** | 1 | Gallego-Martinez 2020 | - |
| ***VCAM1*** | 1 | - | Gallego-Martinez 2020 |
| ***VCL*** | 1 | Gallego-Martinez 2020 | - |
| ***VHL*** | 1 | Li 2017 | - |
| ***WBP2*** | 1 | Roman-Naranjo 2020 | - |
| ***WFS1*** | 4 | Gallego-Martinez 2019, Li 2017, Roman-Naranjo 2020 | Frykholm 2006 |
| ***WHRN*** | 2 | Gallego-Martinez 2019, Roman-Naranjo 2020 | - |
| ***WIPF1*** | 1 | - | Gallego-Martinez 2020 |
| ***WNK2*** | 1 | Sun 2018 | - |
| ***WNK4*** | 1 | Sun 2018 | - |
| ***WNT3*** | 1 | - | Gallego-Martinez 2020 |
| ***WNT4*** | 1 | - | Gallego-Martinez 2020 |
| ***WNT5A*** | 1 | - | Gallego-Martinez 2020 |
| ***WNT5B*** | 1 | Gallego-Martinez 2020 | - |
| ***WNT7A*** | 1 | - | Gallego-Martinez 2020 |
| ***WNT7B*** | 1 | - | Gallego-Martinez 2020 |
| ***XIST*** | 1 | Sun 2018 | - |
| ***XK*** | 1 | Sun 2018 | - |
| ***XKR3*** | 1 | Sun 2018 | - |
| ***YWHAEP7*** | 1 | Sun 2018 | - |
| ***ZBED2*** | 1 | Mehrjoo 2020 | - |
| ***ZF*** | 1 | - | Vrabec 2008 |
| ***ZFP57*** | 1 | Sun 2018 | - |
| ***ZFY*** | 1 | Sun 2018 | - |
| ***ZSCAN23*** | 1 | Sun 2018 | - |
